# Mobility restrictions were associated with reductions in COVID-19 incidence early in the pandemic: evidence from a real-time evaluation in 34 countries

**DOI:** 10.1038/s41598-021-92766-z

**Published:** 2021-07-02

**Authors:** Juhwan Oh, Hwa-Young Lee, Quynh Long Khuong, Jeffrey F. Markuns, Chris Bullen, Osvaldo Enrique Artaza Barrios, Seung-sik Hwang, Young Sahng Suh, Judith McCool, S. Patrick Kachur, Chang-Chuan Chan, Soonman Kwon, Naoki Kondo, Van Minh Hoang, J. Robin Moon, Mikael Rostila, Ole F. Norheim, Myoungsoon You, Mellissa Withers, Mu Li, Eun-Jeung Lee, Caroline Benski, Sookyung Park, Eun-Woo Nam, Katie Gottschalk, Matthew M. Kavanagh, Thi Giang Huong Tran, Jong-Koo Lee, S. V. Subramanian, Martin McKee, Lawrence O. Gostin

**Affiliations:** 1 Department of Social and Behavioral Sciences, Harvard T.H. Chan School of Public Health, 677 Huntington Avenue, Boston, MA 02115 USA; 2 Harvard Center for Population & Development Studies, 9 Bow st, Cambridge, MA 02138 USA; 3 Department of Global Health and Population, Harvard T.H.Chan School of Public Health, 677 Huntington Avenue, Boston, MA 02115 USA; 4 Seoul National University College of Medicine, 103 Daehak-ro, Jongno-gu, Seoul, Republic of Korea; 5 Institute of Convergence Science, Convergence Science Academy, Yonsei University, 50 Yonsei-ro Seodaemun-gu, Seoul, 03722 Republic of Korea; 6 Hanoi University of Public Health, 1A Duc Thang Road, Duc Thang Ward, North Tu Liem District, Hanoi, Vietnam; 7 Boston University School of Medicine, 72 East Concord St., Boston, MA 02118 USA; 8 The University of Auckland School of Population Health, Private Bag 92019, Auckland, 1142 New Zealand; 9 The University of the Americas, Av. Manuel Montt 948, Providencia, Región Metropolitana Santiago Chile; 10 Department of Public Health Sciences, Graduate School of Public Health, Seoul National University, 1 Gwanak-ro, Gwanak-gu, Seoul, 08826 Republic of Korea; 11 Mailman School of Public Health, Columbia University, 722 West 168th St., New York, NY 10032 USA; 12 National Taiwan University College of Public Health, Institute of Environmental and Occupational Health Sciences, 17 Xuzhou Road, Taipei, Taiwan; 13 Kyoto University School of Public Health, Yoshida-konoecho, Sakyo-ku, Kyoto, 606-8501 Japan; 14 City University of New York Graduate School of Public Health and Health Policy, 55 W 125th St, New York, NY 10027 USA; 15 Stockholm University, 106 91 Stockholm, Sweden; 16 University of Bergen, P.O. Box 7800, 5020 Bergen, Norway; 17 University of Southern California, Trousdale Pkwy, Los Angeles, CA 3551 USA; 18 Sydney School of Public Health, The University of Sydney, Sydney, NSW 2006 Australia; 19 Freie Universität Berlin, Otto-von-Simson Str. 11, 14195 Berlin, Germany; 20 University Hospital of Geneva, Boulevard de la Cluse 30, 1205 Genève, Suisse; 21 National Health Insurance Service , 32 Geongang-ro, Wonju, Gangwon-do Republic of Korea; 22 Yonsei University, 1 Yonseidae-gil, Gangwon-do, Wonju, 220-710 Republic of Korea; 23 Georgetown University, 37th and O St., N.W., Washington, DC 20057 USA; 24 Hanoi Medical University, No 1, Tong Tung Street, Hanoi, 116001 Vietnam; 25 London School of Hygiene and Tropical Medicine, 15-17 Tavistock Place, London, WC1H 9SH UK

**Keywords:** Diseases, Health care

## Abstract

Most countries have implemented restrictions on mobility to prevent the spread of Coronavirus disease-19 (COVID-19), entailing considerable societal costs but, at least initially, based on limited evidence of effectiveness. We asked whether mobility restrictions were associated with changes in the occurrence of COVID-19 in 34 OECD countries plus Singapore and Taiwan. Our data sources were the *Google Global Mobility Data Source,* which reports different types of mobility, and COVID-19 cases retrieved from the dataset curated by *Our World in Data.* Beginning at each country’s 100th case, and incorporating a 14-day lag to account for the delay between exposure and illness, we examined the association between changes in mobility (with January 3 to February 6, 2020 as baseline) and the ratio of the number of newly confirmed cases on a given day to the total number of cases over the past 14 days from the index day (the potentially infective ‘pool’ in that population), per million population, using LOESS regression and logit regression. In two-thirds of examined countries, reductions of up to 40% in commuting mobility (to workplaces, transit stations, retailers, and recreation) were associated with decreased cases, especially early in the pandemic. Once both mobility and incidence had been brought down, further restrictions provided little additional benefit. These findings point to the importance of acting early and decisively in a pandemic.

## Introduction

Imposition of restrictions on mobility have been the cornerstone of policy responses to the novel coronavirus (SARS-CoV-2) pandemic^1^. These restrictions have included both incentives, encouraging working from home, facilitated by expansion of online resources that enable meetings, teaching, and shopping; and sanctions such as stay at home orders, restrictions on travel, and closure of shops, offices, and public transport^2–5^. They are unprecedented in both scale and scope compared to previous epidemic responses^6^.

The rationale underpinning these public health measures is that restricting normal activities decreases the number, duration, and proximity of interpersonal contacts and thus the potential for viral transmission. Transmission simulations using complex mathematical modelling have built on past experience such as the 1918 influenza epidemic^7^, as well as assumptions about the contemporary scale and nature of contact in populations^8^. However, the initial models inevitably depended on the state of knowledge of the new virus, with the crucial role of airborne transmission only later being appreciated, and limited empirical evidence from behavioral scientists on the feasibility or sustainability of mass social and behavior change in contemporary society. While reductions in interpersonal contact and increases in physical distancing are known to decrease spread of respiratory infection^9^, the paucity of recent examples of large-scale restrictions on mobility when dangerous viruses were circulating widely has limited the scope for research on their impact on transmission. Where restrictions have been imposed, as with Ebola, they have involved diseases with a different mode of transmission. Nonetheless, the rapidity of progression of this pandemic has forced many governments into trialing various approaches to containment with limited evidence of effectiveness^10^.

More conventional public health prevention measures (such as quarantine of contacts, isolation of infected individuals and contact tracing) and control measures in health systems (such as patient flow segregation, negative pressure ventilation, and use of personal protective equipment)^11–14^, have been applied widely to control the pandemic in many countries as part of a portfolio of policy responses. However, mobility restriction as a new large-scale mass behavioral and social prescription has incurred considerable costs^15,16^. Estimates suggest global GDP growth has fallen by as much as 10%^17^, at least part of which can be attributed to these mobility restrictions. Although views differ, not least because of the relative lack of information of what would happen if the disease was unchecked (although the devastating situation in India in early 2021 does provide some insight) and the initial inadequate recognition of persisting disability in a substantial number of survivors (Long COVID), at the beginning of the pandemic some expressed concern that the economic damage of restrictions might be greater than would be accounted for by the impact of direct illness and deaths from COVID-19^18,19^. However, there are now some studies that do find a link between mobility restrictions and reductions in viral transmission^20^, while a recent analysis of GDP and death rates finds that poorer pandemic control tends to be associated with greater economic loss^21^.

Even though the pandemic continues, those calling for a rapid easing of restrictions are becoming increasingly vocal. Hence, there is an urgent need to assess the effectiveness of decisions to apply large scale restrictions of mobility in limiting pandemic spread. To this end, we examined the association of mobility with COVID-19 risk in Organization of Economic Cooperation and Development (OECD) countries and the equivalent economies, Singapore and Taiwan.

## Methods

### Study population and data

The study population consisted of the populations of 36 countries (all 34 OECD countries plus Singapore and Taiwan). Two sets of publicly available data were pooled into one dataset: country-specific newly confirmed COVID-19 cases per day retrieved from the dataset curated by *Our World in Data* (https://ourworldindata.org/coronavirus)^22^, based on data collected from multiple national sources by the Center for Systems Science and Engineering at Johns Hopkins University, and country-specific mobility change data obtained from the *Google Global Mobility Data Source*^23,24^.

### Variables

#### Independent variables

We used country-specific mobility data, collected from mobile device-based global positioning system (GPS) information from people who agreed to share their anonymized position information with Google, starting from the date the 100th case was detected in a country. The average mobility around specific categories of locations during the reference period (between January 3 and February 6, 2020, a period before COVID-19 was declared a global pandemic and the earliest period for which data are available) was applied as a baseline against which changes were calculated. The data were categorized by Google into 6 different types of locations visited (workplaces, transit stations, retailer and recreational places, residential areas, groceries and pharmacies, and parks). To reduce multicollinearity, three highly correlated variables (workplace mobility, transit station mobility, and retailer and recreation mobility) were averaged into a combined latent variable (“commuting mobility”) as the main variable for this study, as this is the activity that is most likely to bring large groups of people together (Supplementary Fig. 1).

#### Outcome variables

The outcome variable chosen was the ratio of the number of new cases on a given day to the total number of cases over the past 14 days from the index day (the potentially infective ‘pool’ in that population), per 100,000 population, starting from the 14th day from the date the 100th case was detected in each country through August 31, 2020.

### Analyses

We examined country-specific associations between the percentage change in mobility for each of the 36 countries and the ratio of new COVID-19 cases to the total infective pool numbers, with a 14-day lag, using LOESS (Locally Weighted Scatter-plot Smoother) regression, a nonparametric technique that uses locally weighted regression to fit a smooth curve between an outcome and predictor variable(s) through points in a scatter plot. The fitting at point x is weighted toward the data nearest to X^25^. Based on the range and frequency of commuting mobility values observed across countries, we grouped values into mild (up to 20%), moderate (21–40%) and extensive (greater than 40%) degrees of mobility change. Logit-transformed ratio were regressed to mobility changes to compare the country-specific association using a single regression coefficient for each country. We performed additional analysis by designating an early phase versus late phase using the median date (between the day of the 100th case and the last day of study period–August 31) for each country; and by using different mobility location categories (i.e. residential areas, parks, or groceries and pharmacies) beyond the main analysis focused on commuting mobility. The unit of analysis was country level. All analyses used R (version 4.0.0, R Foundation for Statistical Computing, Vienna, Austria).

#### Sensitivity analyses

We performed sensitivity analyses by varying the lag interval (7 or 21 days) between the change in mobility and ratio of new cases to infective pool numbers, to take account of variations in incubation periods.

## Results

**Figure 1:**
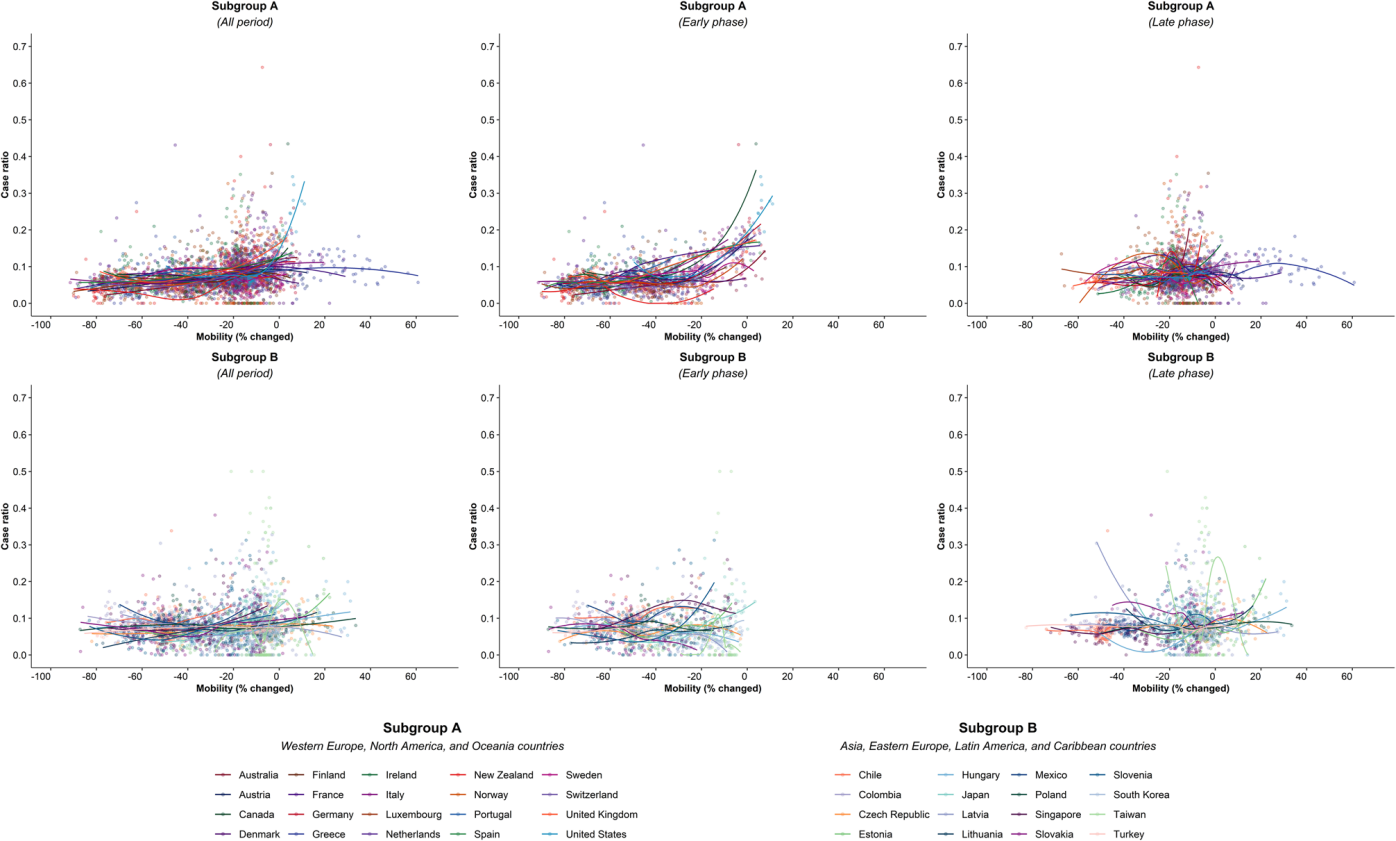
Association between COVID-19 case ratio and mobility changes for 36 countries by pandemic phase, arbitrarily split by geography to allow better resolution of the data. Footnote 1. The mobility change measurement period was from the day of the 100th case in each country through August 31, 2020. Footnote 2. Pandemic phase was defined for each country by the median of the date when the 100th case was detected to the end of the study period: early phase for the period before the median date and late phase for the period after the median date.

**Figure 2:**
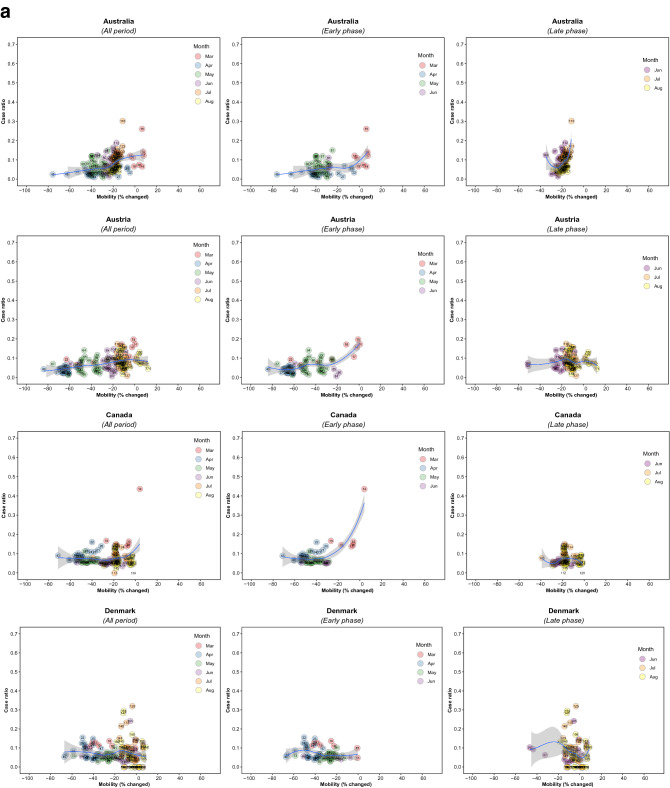

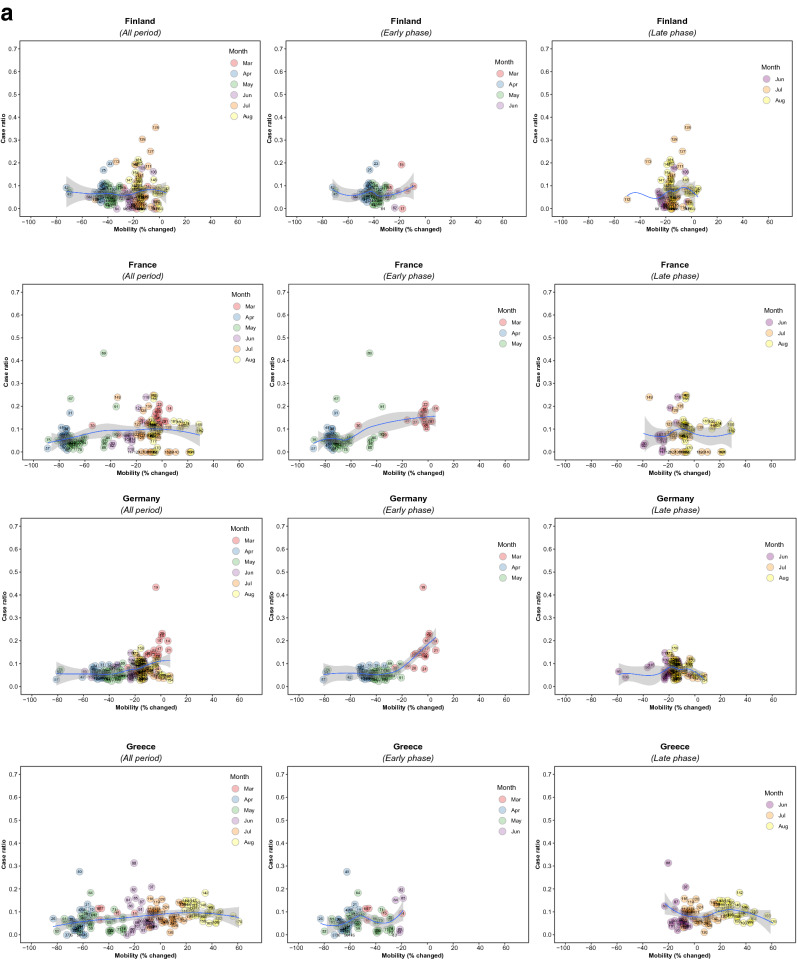

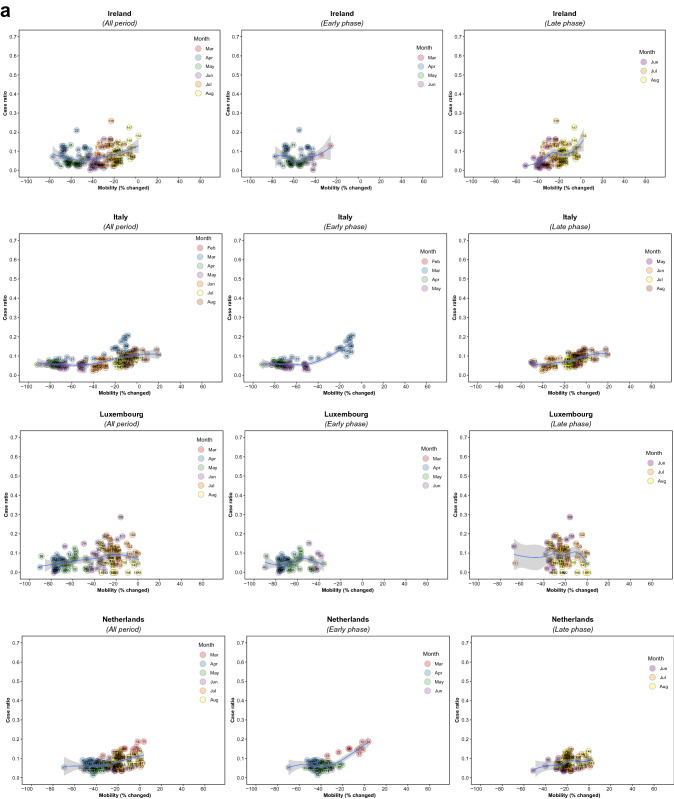

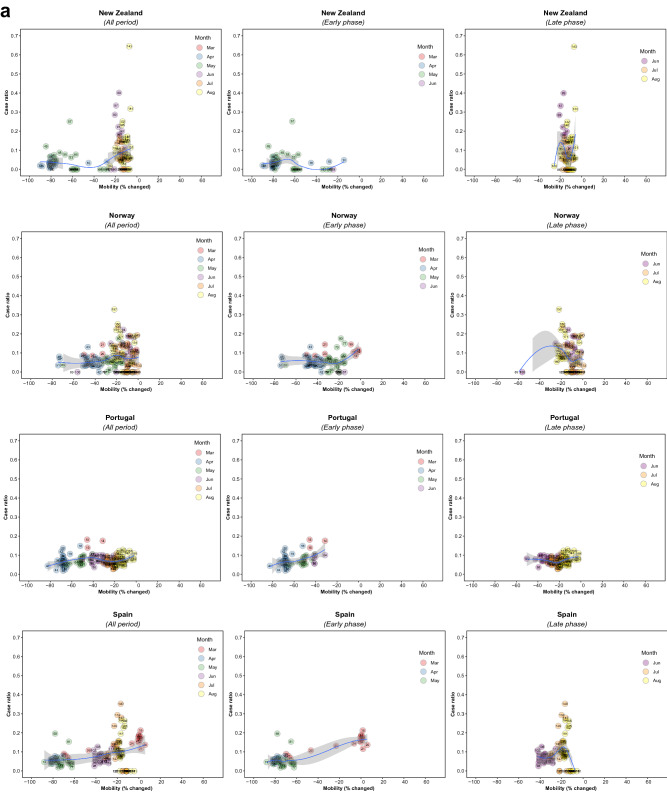

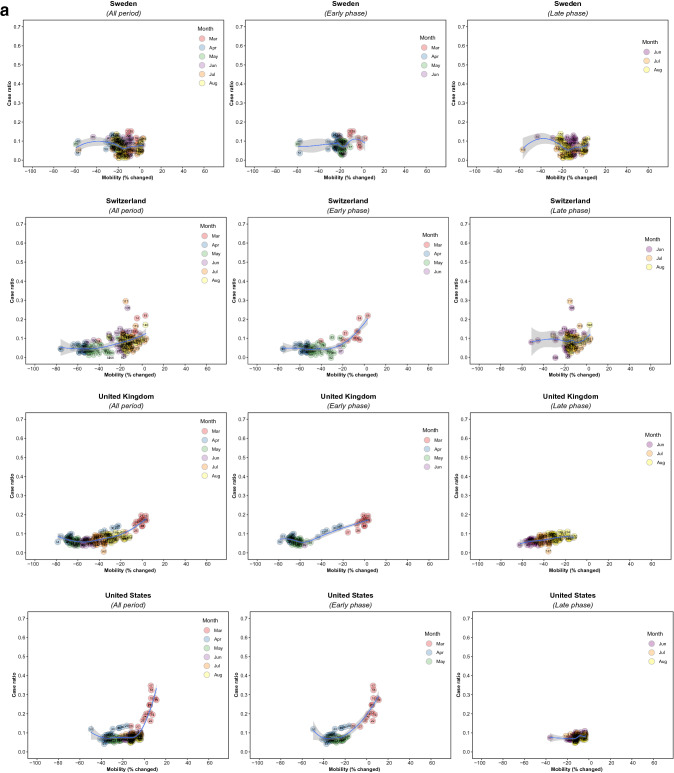

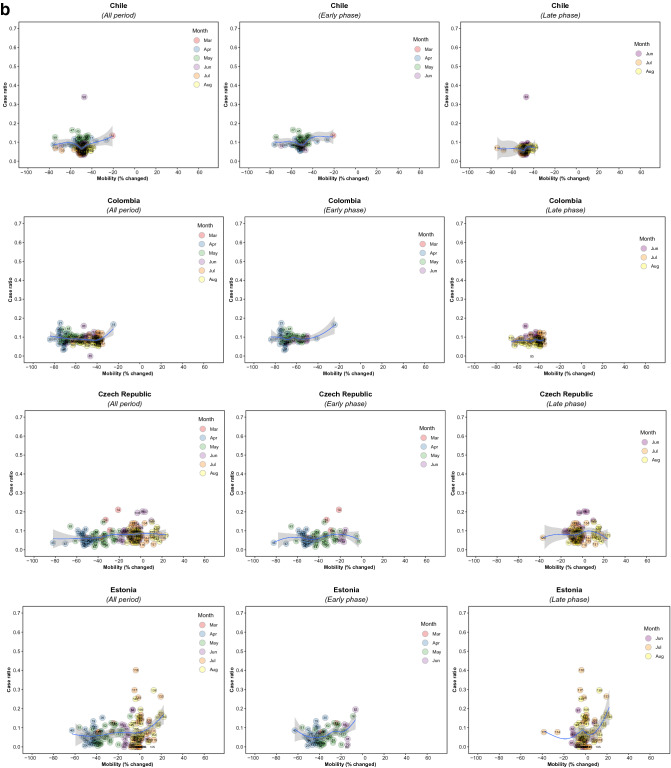

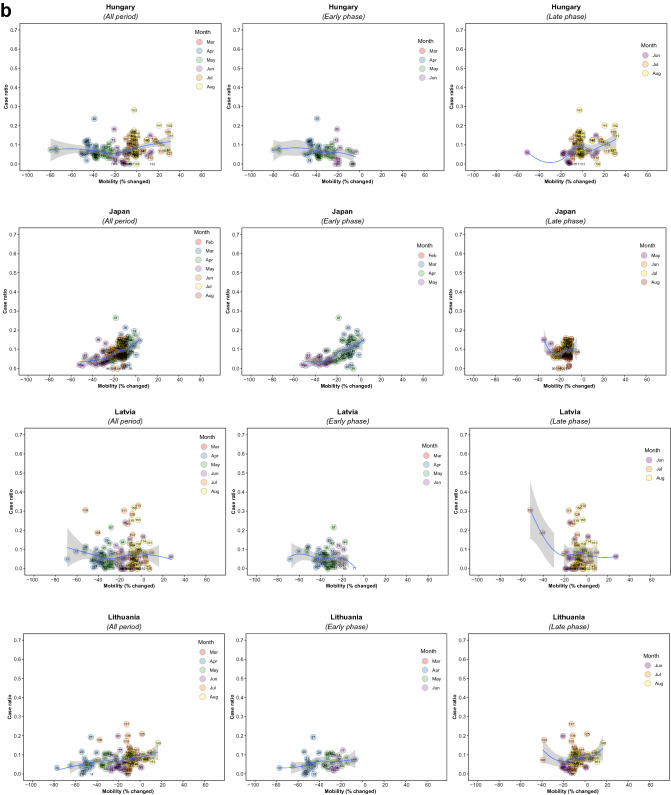

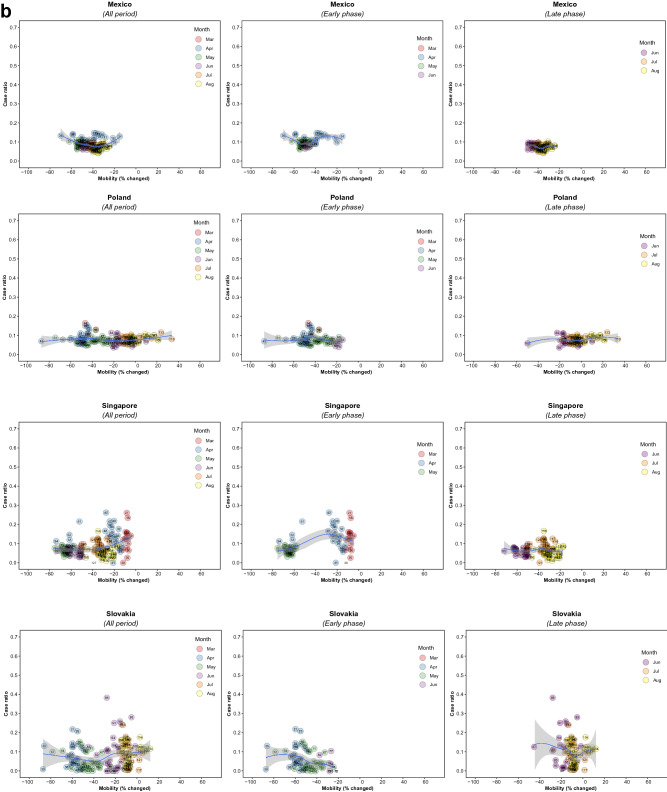

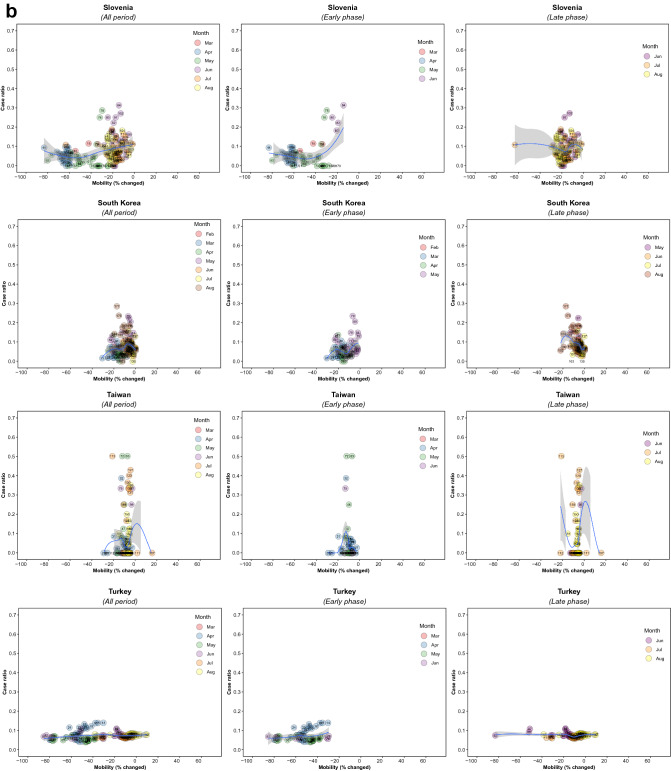
Association between new daily COVID-19 case ratio and commuting mobility changes in each of 36 countries by pandemic phase. (**A**) Western Europe, North America, and Oceania countries. (**B**) Asia, Eastern Europe, Latin America, and Caribbean countries. Footnote 1. The mobility change measurement period was from the day of the 100th case in each country through August 31, 2020. Footnote 2. Pandemic phase was defined for each country by the median of the date when the 100th case was detected to the end of the study period: early phase for the period before the median date and late phase for the period after the median date.

**Figure 3:**
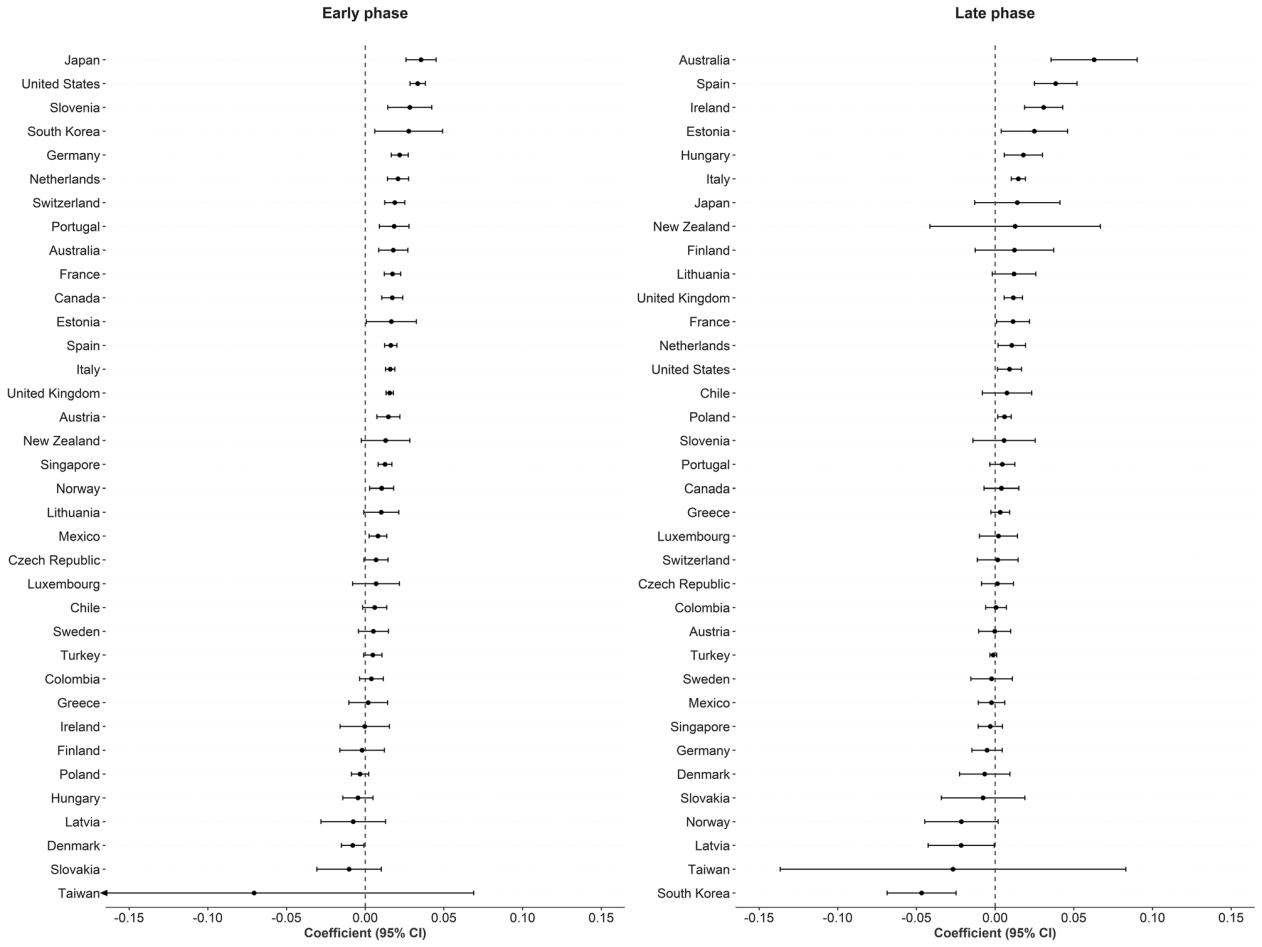
Forest plot showing unadjusted estimate for the association of COVID-19 case ratio logit-transformed with mobility changes. Footnote 1. The mobility change measurement period was from the day of the 100th case in each country through August 31, 2020. Footnote 2. Pandemic phase was defined for each country by the median of the date when the 100th case was detected to the end of the study period: early phase for the period before the median date and late phase for the period after the median date.

There were 5,766 observations with data on daily COVID-19 infections case ratio and mobility changes from 36 countries, starting with the 100th case in each country, which ranged from February 21 (South Korea) to March 23 (New Zealand) and continuing until August 31, 2020. The changes in commuting mobility were pronounced, from a 91.8% decrease (Spain on Apr 10) to a 60.8% increase (Greece on Aug 16). The national daily COVID-19 cases ratio was as high as 0.64 (New Zealand on Mar 10). There was a reduction in most countries with mild (up to 20%) to moderate (21–40%) decrease in commuting mobility but, in most cases, then reached a plateau with little further change with extensive (>40%) decreases in mobility (Figs. 1 and 2). The country- and phase-specific slopes of the curves (point estimation with 95% confidence interval) are shown in Fig. 3. The association was stronger in the early phase. A statistically significant positive association between mobility change and case ratio was seen in 19 countries in the early phase while it was present in only 11 countries in the late phase.

**Figure 4:**
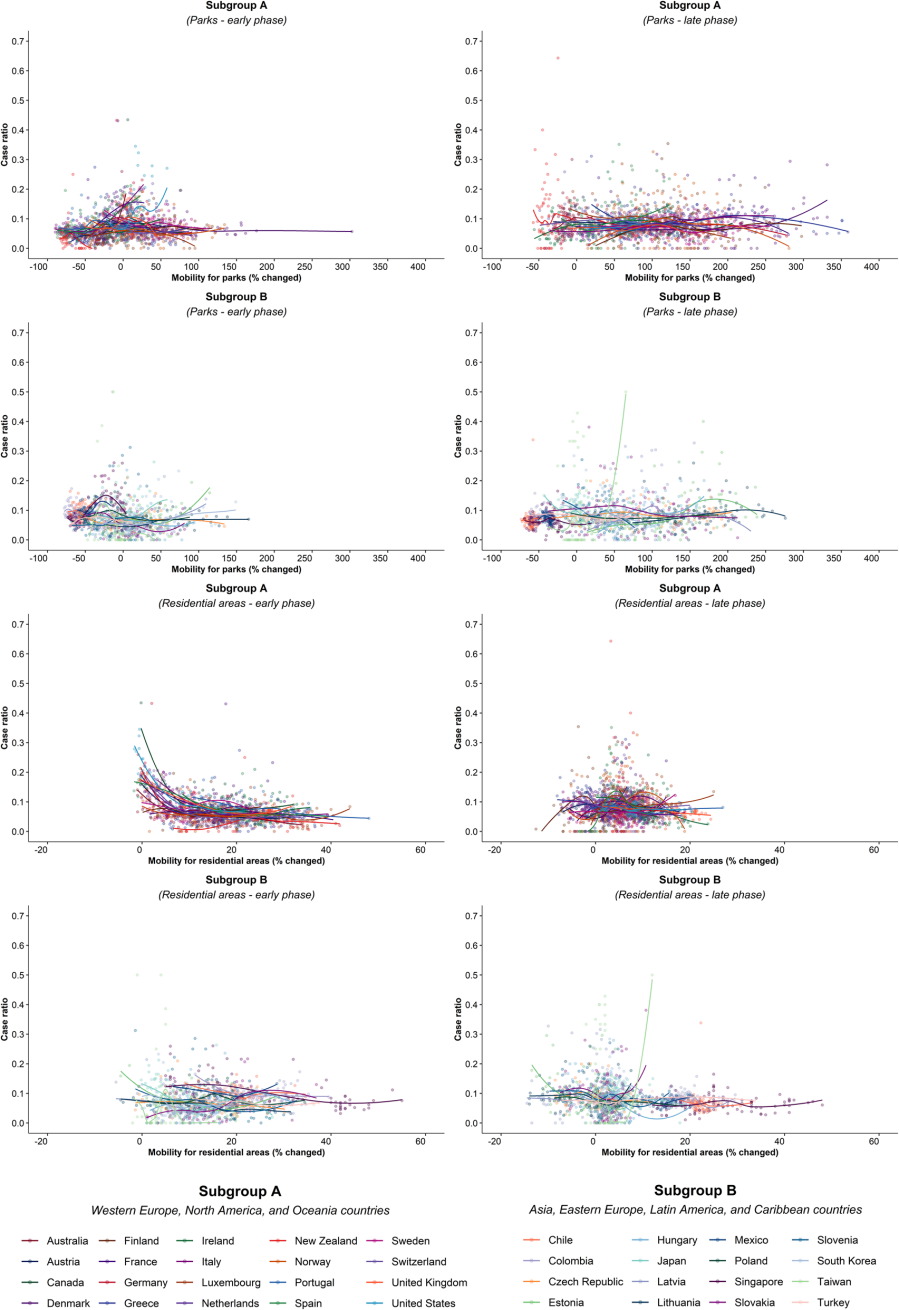
Association between new daily COVID-19 case ratio and mobility changes for 36 countries, early and late phase, for parks and residential areas. Footnote 1. Pandemic phase was defined for each country by the median of the date when the 100th case was detected to the end of the study period: early phase for the period before the median date and late phase for the period after the median date.

Of note, small increases in mobility in residential areas, seen throughout most of the study period in all countries, were associated with reductions in COVID-19 ratio, but larger increases had no additional effect in the early phase, and there were no associations in any ranges in the late phase (Fig. 4; supplementary Fig. 2). In the early phase, increased mobility in parks was associated with increased incidence in 5 countries (US. Spain, Japan, Estonia, and Latvia) whereas it was not associated in other countries. In the late phase, there was no association in most countries (Supplementary Fig. 2). Mobility around groceries and pharmacies, generally viewed as essential visits, was typically associated with the highest incidence of infection at a time when mobility was close to baseline, during the early phase (Supplementary Fig. 3). In the late phase, however, this finding no longer held and there was no association between infection case ratio and mobility, regardless of the degree of mobility change (Supplementary Fig. 3). Overall, results from the late phase show little association between case ratio and mobility changes in any locations in most countries, except some for a reduction in ratio with moderate restriction in commuting mobility in Ireland, Australia, Italy, Spain, Estonia, and Hungary; and an increased incidence with increased mobility around parks in Estonia, Hungary, and Spain (Figs. 1, 2, 3, and 4; Supplementary Figs. 3 and 4). The findings were mostly consistent in the sensitivity analysis when we used alternative lag periods of 7 or 21 days in the model (Supplementary Fig. 4).

## Discussion

Overall, the expectation that reduced mobility would be associated with reduced occurrence of new cases of COVID-19 was confirmed, with a minimum reduction of 20–40%. However, the decrease plateaued as mobility remained low or decreased further. Mobility restrictions were most effective in the early phase of the pandemic, with a much smaller effect in the later phase.

This study has several limitations. First, Google data are only generated by Android users who have location services switched on. These individuals may be an atypical minority of the population in some countries, or there may be changes in the type of users over time, such as tourists (as in Greece), although in most cases the impact will be small. Second, Google only published data from early 2020 and, as mobility is seasonal, it would have been preferable to have used the same week in previous years as baseline. Third, the number of cases is influenced by the availability of testing and quality of reporting. Fourth, the use of an ecological design introduces scope for confounding and imprecision: our data do not allow us to isolate the impact of mobility restrictions from the many other variables that intervene in a pandemic response, such as the degree of inter-household mixing, the ability to detect and rapidly control an outbreak, and behavioral characteristics such as use of face coverings and adherence to physical distancing guidelines, themselves influenced by clarity of messaging and trust in official advice. A related limitation is that, in large and, especially, federal countries such as the United States, there may be substantial sub-national differences in implementation of these characteristics and in other policies. However, given the complex nature of these relationships, influenced by starting conditions, feedback loops, and non-linear relationships, the analytic challenges of disentangling these factors are formidable even if data were available.

There are a variety of possible explanations for our findings. Mandatory restrictions on mobility may reduce both the frequency and/or duration of interpersonal interactions and, by reinforcing official advice, the nature of those interactions, with greater distancing and hygiene precautions. As restrictions are lifted, protective behaviors such as distancing may have become normalized, mitigating the effects of greater mobility. When this is coupled with improved contract-tracing, quarantine, and support for isolation, the relative benefits of mobility restrictions may decrease as the population adopts additional risk mitigation measures, allowing a return to greater mobility (adapting to the “new normal”). These behavioral changes may also be enhanced through social learning of these practices promoted by trusted and authoritative public health messaging. Should clear and consistent messaging be discontinued it is also possible that reduced adherence to restrictions could further attenuate their impact. While our findings support the role of mobility restrictions as a critical strategy early in a pandemic or when a country has difficulty implementing more comprehensive and meticulous mitigation strategies, they also suggest that rapid scale-up of a suite of other simultaneous coordinated mitigation schemes as part of a strategy to drive transmission as low as possible may maximize health gains while enabling a gradual reopening of the economy^26–31^.

These findings are consistent with other research suggesting benefits of early mobility restrictions^20^; for example, an earlier study of government-imposed physical distancing interventions demonstrated an association with larger reductions in COVID-19 incidence, although this did not directly assess the degree of mobility reduction nor the impact of timing of the interventions^32^. Modeling in China using mobile phone data in the initial phase of the pandemic provides further support for this view^33^.

The Google data use mobile phone tracking to analyze real-time changes in mobility. In at least some jurisdictions the public seems to have pre-empted official instructions, reacting to emerging data^34^. One study in the United States, where implementation of other public health mitigation strategies has varied, found a strong correlation between decreased mobility patterns and lower COVID-19 case growth rates using similar phone tracking data^34^.

Our analysis focused on changes in commuting mobility. However, this is only one way in which people mix and spread infections, with inter-household spread important in some settings^35^. Although we did find some associations between incidence and, for example, mobility in residential areas, these are difficult to interpret and investigation of these phenomena will require other data sources, such as apps that record close contacts. Finally, our finding of little difference using different lag times (7, 14 or 21 days) is consistent with prior research suggesting a typical lag between interventions and changes in infection rate of around 14 days^34,36^.

While additional work is needed to elaborate on these findings, they may be useful to policymakers as they adapt responses to the pandemic over time and, in particular, point to the importance of including mobility data when developing comprehensive strategies where this is not already happening.

Severe restrictions on mobility, or lockdowns, may be blunt but are necessary instruments at the beginning of a pandemic. If sufficiently stringent to get the reproductive number below 1, such restrictions will drive down the level of infection but at considerable human and economic cost. The time bought must be used to establish effective testing and tracing systems. If both are done, and other non-pharmacological interventions such as wearing of face coverings and social distancing are adhered to, it should then be possible to slowly open up society in the knowledge that local resurgences can be contained. However, if these systems fail and cases rise rapidly, mobility restrictions may be required again, but only as a last resort and seen as likely evidence of policy failure.

Real-world observations after this empirical analysis, especially during October 2020 and January 2021, support our speculation. We can identify certain characteristics of countries with successful responses. First, those that controlled their borders, with temporary isolation of infective people using public health measures such as isolation and quarantine coupled with contact-tracing and testing were able to have less strict or minimal mobility restrictions for the general public as there was a very low probability of transmission among the general public beyond isolation facilities. The countries that were closest to this ideal environment were New Zealand, Taiwan, and Singapore. These have enjoyed near normal mobility and avoided major outbreaks during the large surge in other countries in Oct 2020 to Jan 2021. Second, if a jurisdiction has not ensured temporary isolation of infective people sufficiently, there should be a positive association between mobility changes in the public and COVID incidence. However, if this jurisdiction maintained low mobility to complement a suboptimal environment with respect to isolation, the jurisdiction might still be able to prevent surge of transmissions. This second typology jurisdiction may enjoy low incidence with the sacrifice of mobility such as Australia. Thirdly, if people in a jurisdiction have embraced individual behavioral prevention protocols with or without strong societal isolation, which allows some people who are infective to move among general public, the association of mobility and incidence might have been near nullified over time. This typology could lead to no or small outbreak only, with a range of mobility recovery during surges in other countries. Examples of this typology include Norway, Finland, Canada, and Germany. Finally, for jurisdictions which are unable to establish either sufficient isolation capacity or individual behavioral changes, mobility restriction would be one of the primary effective measures remaining. In this environment, mobility restrictions and incidence would have been evident, so lowering mobility would be one of the critical contributions, if any, to prevent further surges. This typology might include the United States, the United Kingdom, Italy, and Spain.

Our study only used data from initial phases of the early waves of the pandemic, and further research is needed to confirm our findings during more recent waves of the pandemic, when improved understanding about the virus and its transmission (such as the role of airborne transmission) has led to changes in public advice. In addition, continued work will be needed on the transmission of emerging and potentially more transmissible variants, effectiveness of a range of mitigations, and improved methods of data collection and analysis that can inform our understanding of human behavior and infection.

## Conclusion

Our analysis extends the understanding of the complex dynamics at play when mobility is restricted at a population level in response to a pandemic caused by a respiratory virus. Societal mobility restrictions appear to have reduced COVID-19 spread in many countries, particularly in the early phase of the waves of the pandemic, but in the late phase, once other mitigation measures have been adopted, the magnitude of impact is attenuated. It is critical for policymakers to consider the effectiveness of mobility restriction in COVID 19 response and the economic impacts imposed on society, especially as this pandemic still appears far from an end globally and with an increasing number of variants, societies may need to adjust to the “new normal” way of life. For this, additional evidence, including the relationship with other non-pharmacological interventions, is needed to fully understand the role of mass restrictions on mobility in containing COVID-19 and future infectious diseases with a similar mode of transmission. As the pandemic progresses, governments must develop strategies that limit the amount of circulating virus and allow rapid responses to further outbreaks. The pandemic has brought enormous changes to working and living, some of which will likely persist even with the advent of multiple vaccines. Surveillance that goes beyond incidence of infection, to include risk factors such as mobility, can only improve our ability to develop effective public health responses.

## Supplementary Information

Supplementary Information.

## Data Availability

All data used are publicly available.
