# Supplementary material for: Mobility restrictions were associated with reductions in COVID-19 incidence early in the pandemic: evidence from a real-time evaluation in 34 countries
Source: Sci Rep. 2021 Jul 2;11:13717. doi: 10.1038/s41598-021-92766-z (PMC8253807; doi:10.1038/s41598-021-92766-z)
Supplement: Supplementary file 1 — Supplementary Information. [file 41598_2021_92766_MOESM1_ESM.pdf]

Supplementary Figure 1. Correlation matrix between six categories of mobility locations: workplaces; transit stations; retailer and recreational places; residential areas; groceries and pharmacies; parks; and commuting (average of workplaces; transit stations; retailer and recreational places)

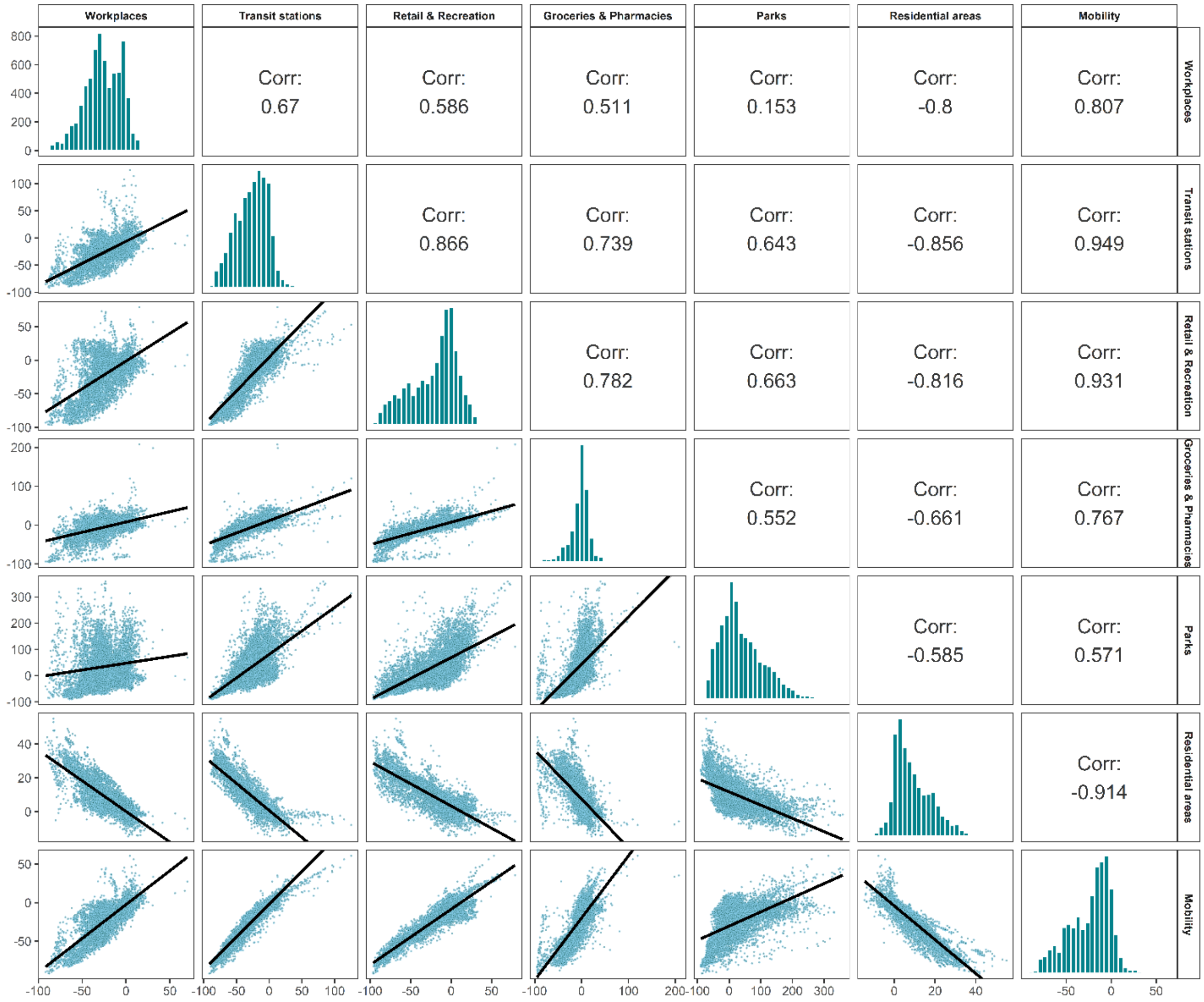

Supplementary Figure 2A. Association between new daily COVID-19 case ratio and mobility changes in 36 countries by early and late phase and other places (parks and residential areas) visited. Western Europe, North America, and Oceania

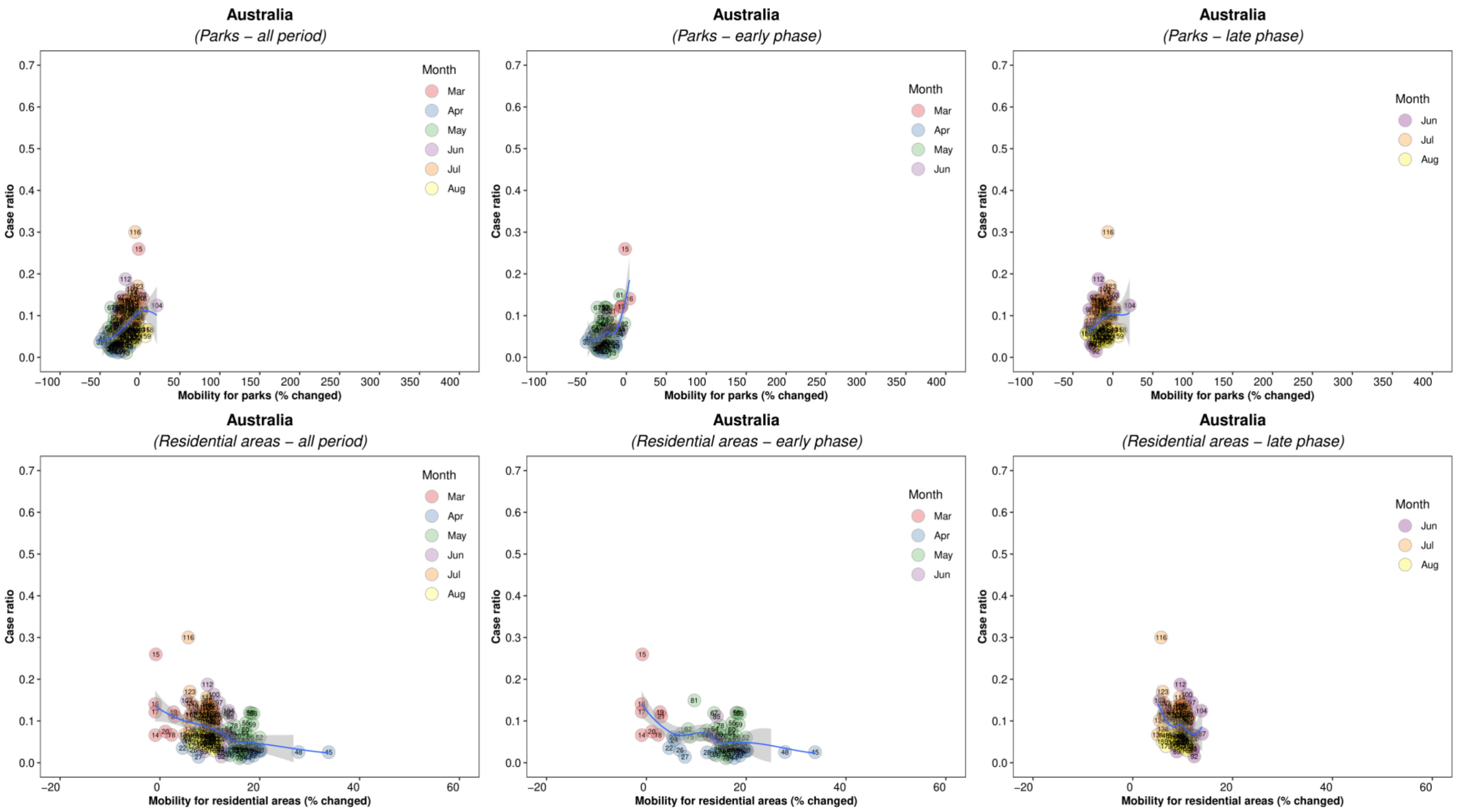

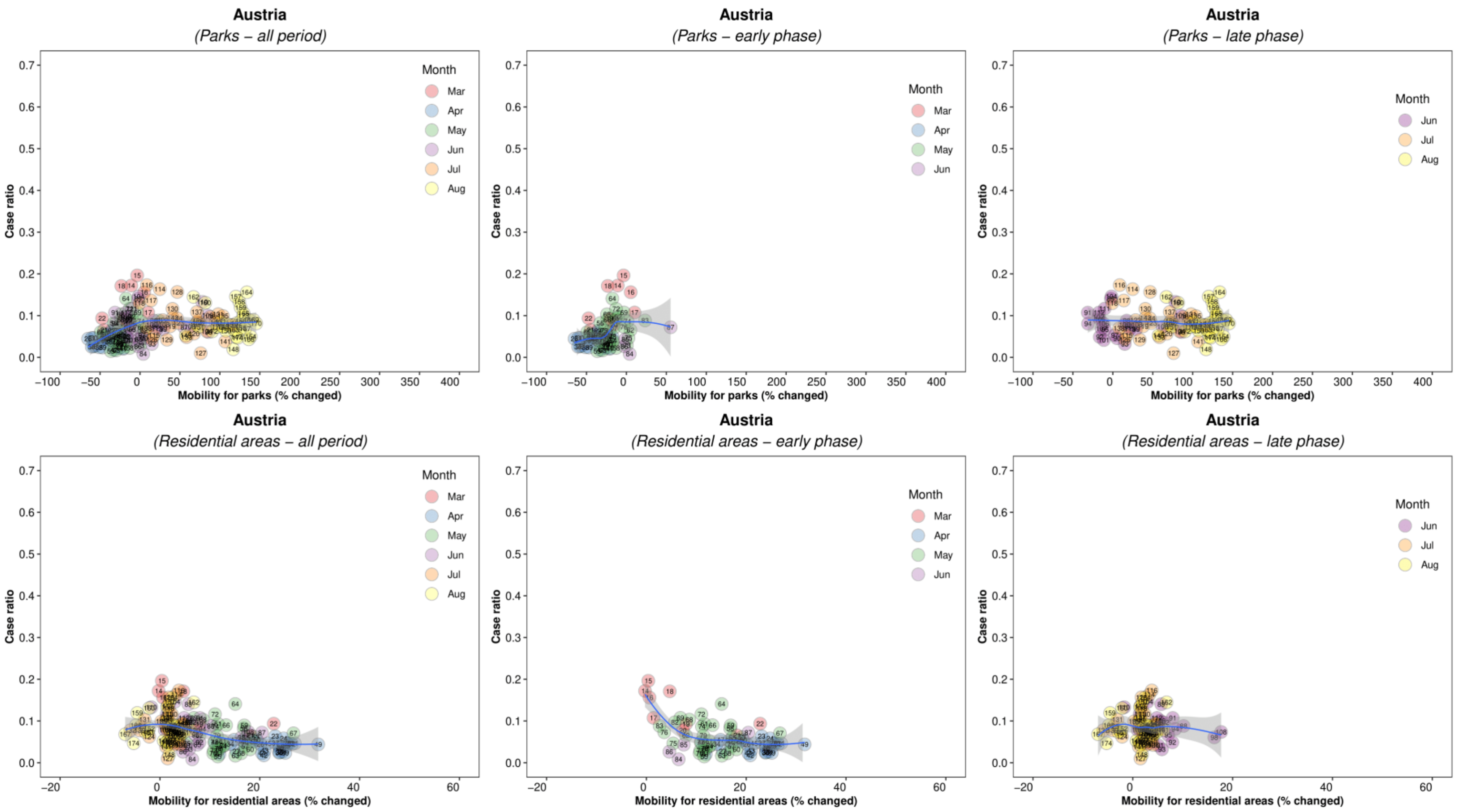
Austria
(Parks – all period)
Austria
(Parks – early phase)
Austria
(Parks – late phase)
Case ratio
Mobility for parks (% changed)
Month
Mar
Apr
May
Jun
Jul
Aug
Austria
(Residential areas – all period)
Austria
(Residential areas – early phase)
Austria
(Residential areas – late phase)
Mobility for residential areas (% changed)

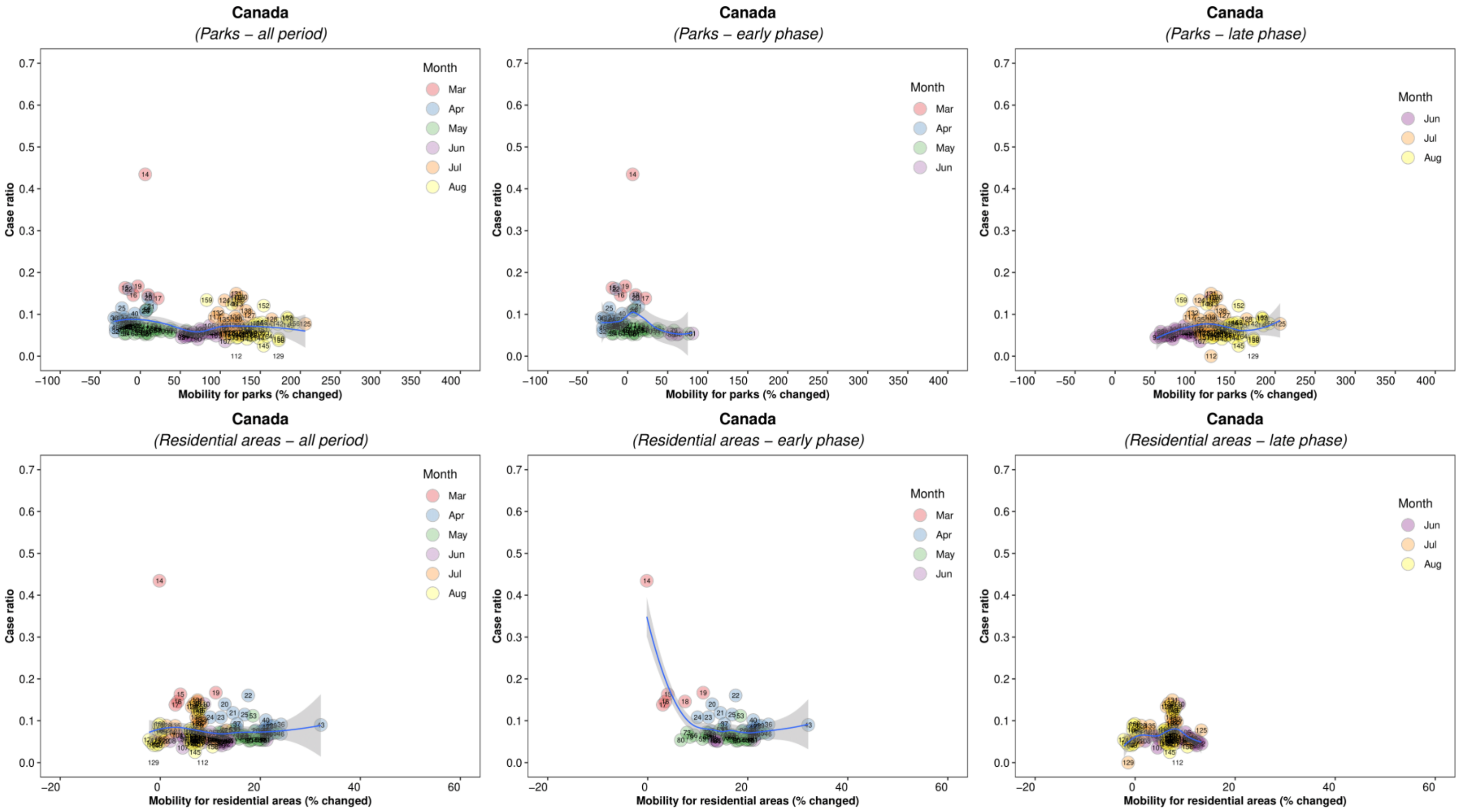

Canada
(Parks – all period)
Canada
(Parks – early phase)
Canada
(Parks – late phase)
Canada
(Residential areas – all period)
Canada
(Residential areas – early phase)
Canada
(Residential areas – late phase)
Case ratio
Mobility for parks (% changed)
Mobility for residential areas (% changed)
Month
Mar
Apr
May
Jun
Jul
Aug

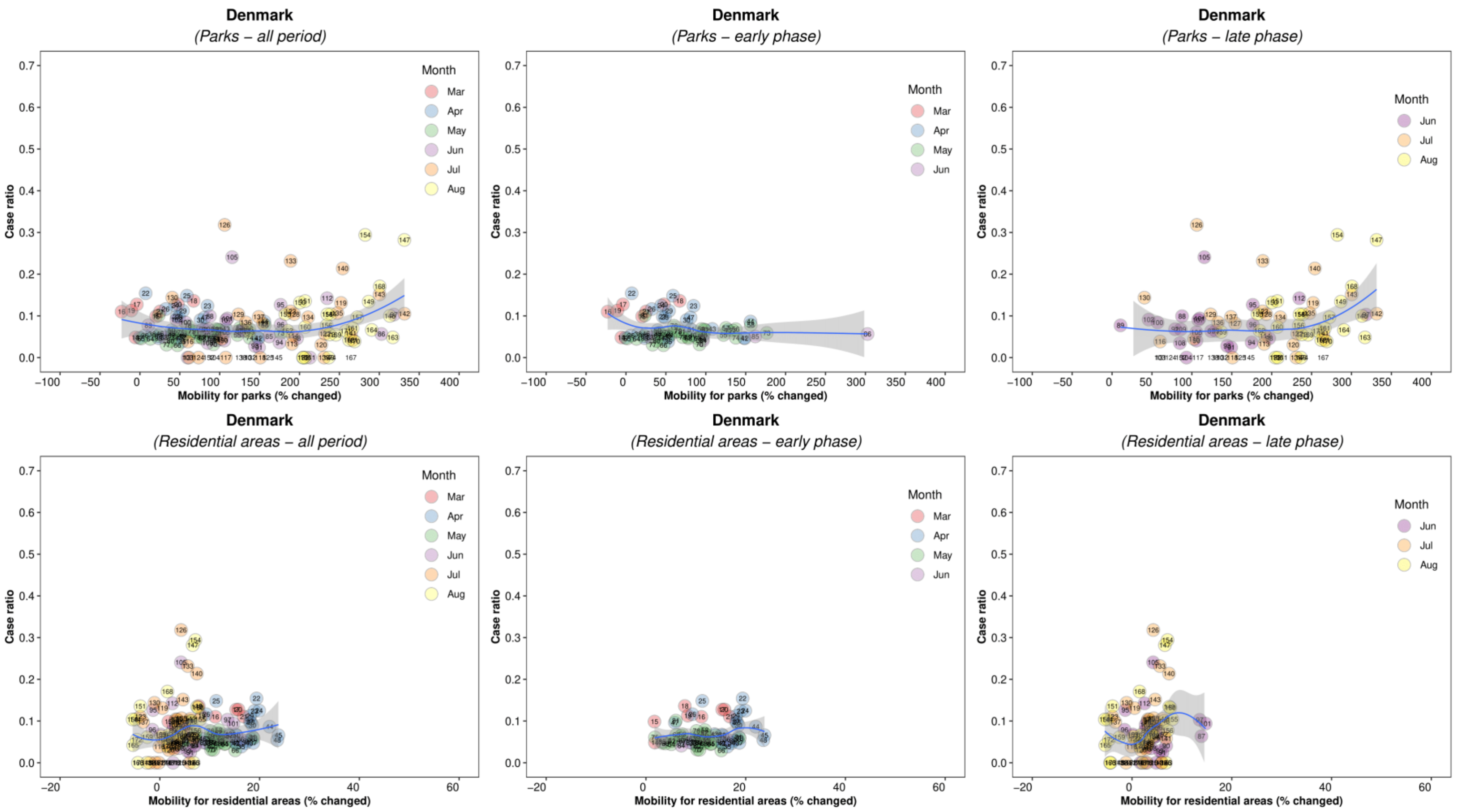
Denmark
(Parks – all period)
Denmark
(Parks – early phase)
Denmark
(Parks – late phase)
Denmark
(Residential areas – all period)
Denmark
(Residential areas – early phase)
Denmark
(Residential areas – late phase)
Case ratio
Mobility for parks (% changed)
Mobility for residential areas (% changed)
Month
Mar
Apr
May
Jun
Jul
Aug

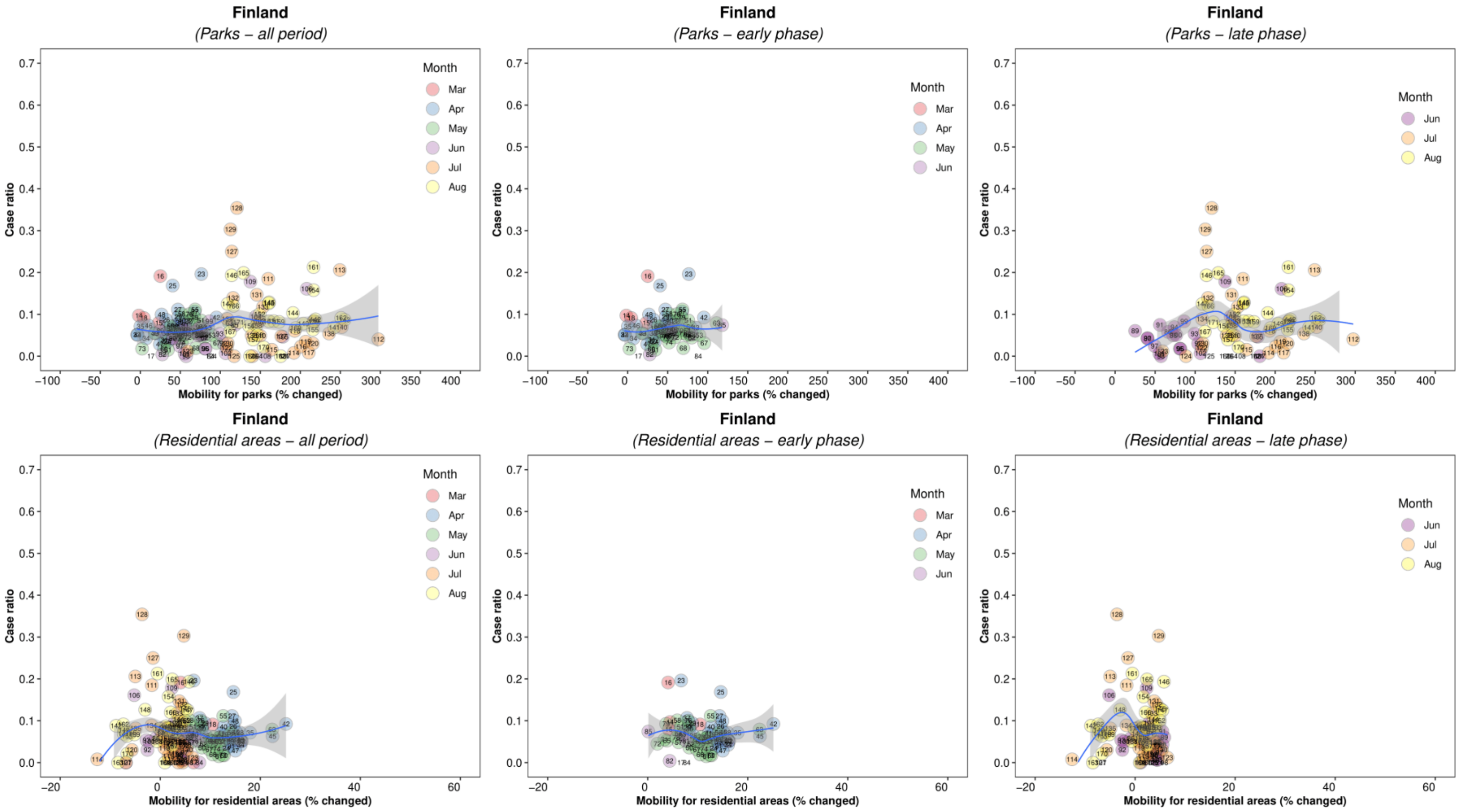
Finland
(Parks – all period)
Finland
(Parks – early phase)
Finland
(Parks – late phase)
Finland
(Residential areas – all period)
Finland
(Residential areas – early phase)
Finland
(Residential areas – late phase)
Case ratio
Mobility for parks (% changed)
Mobility for residential areas (% changed)
Month
Mar
Apr
May
Jun
Jul
Aug

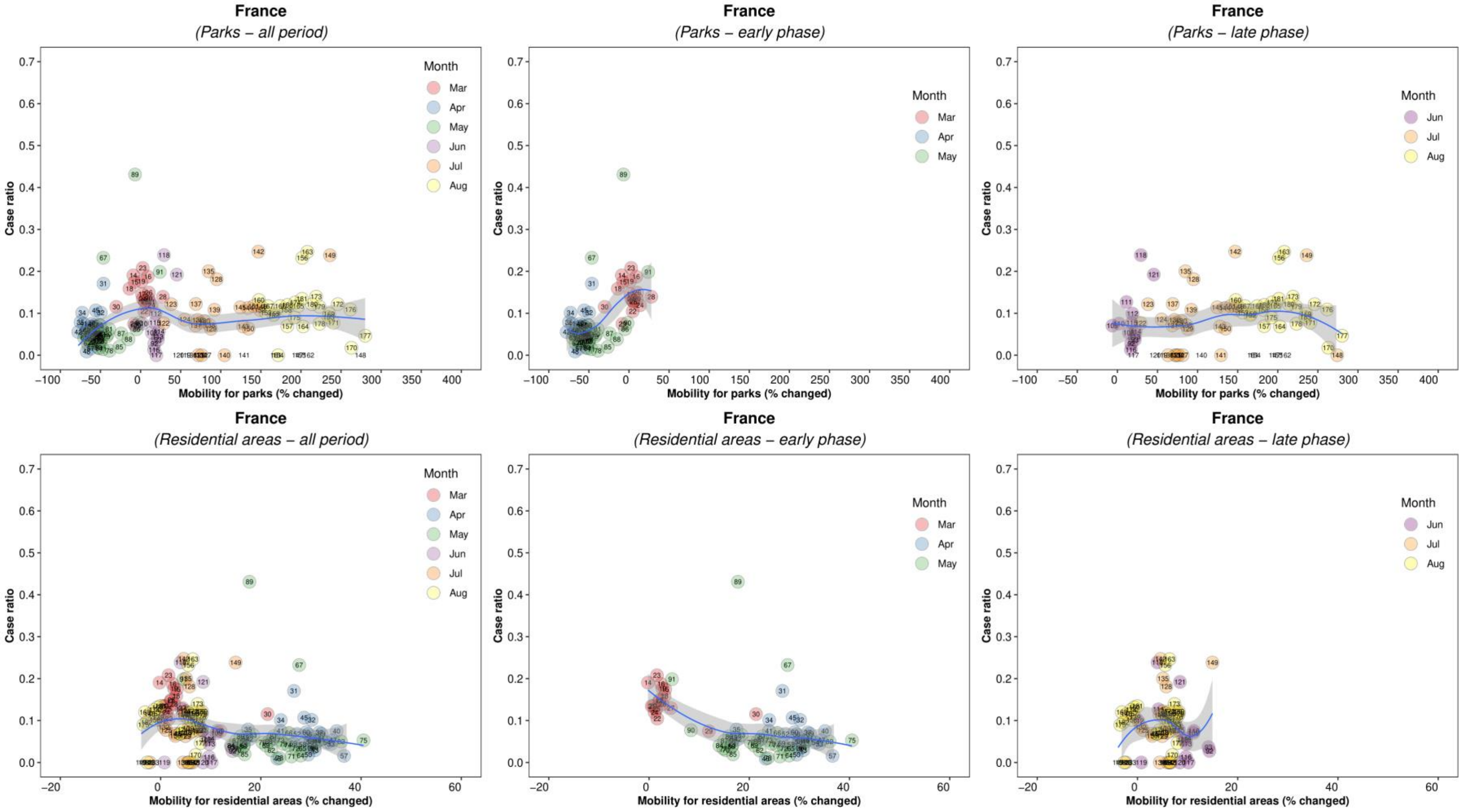
France
(Parks – all period)
France
(Parks – early phase)
France
(Parks – late phase)
France
(Residential areas – all period)
France
(Residential areas – early phase)
France
(Residential areas – late phase)
Case ratio
Mobility for parks (% changed)
Mobility for residential areas (% changed)
Month
Mar
Apr
May
Jun
Jul
Aug

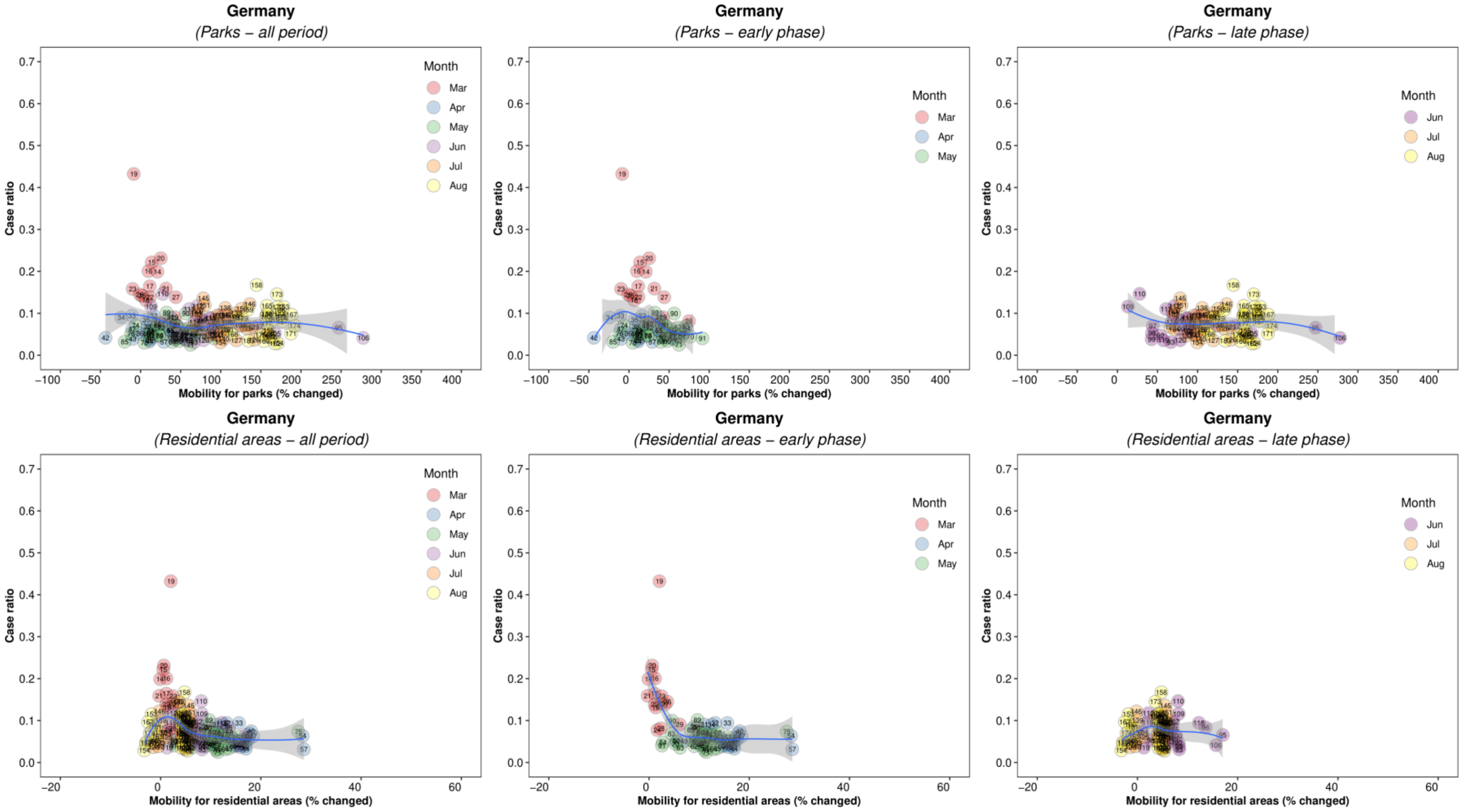
Germany
(Parks – all period)
Germany
(Parks – early phase)
Germany
(Parks – late phase)
Germany
(Residential areas – all period)
Germany
(Residential areas – early phase)
Germany
(Residential areas – late phase)
Case ratio
Mobility for parks (% changed)
Mobility for residential areas (% changed)
Month
Mar
Apr
May
Jun
Jul
Aug

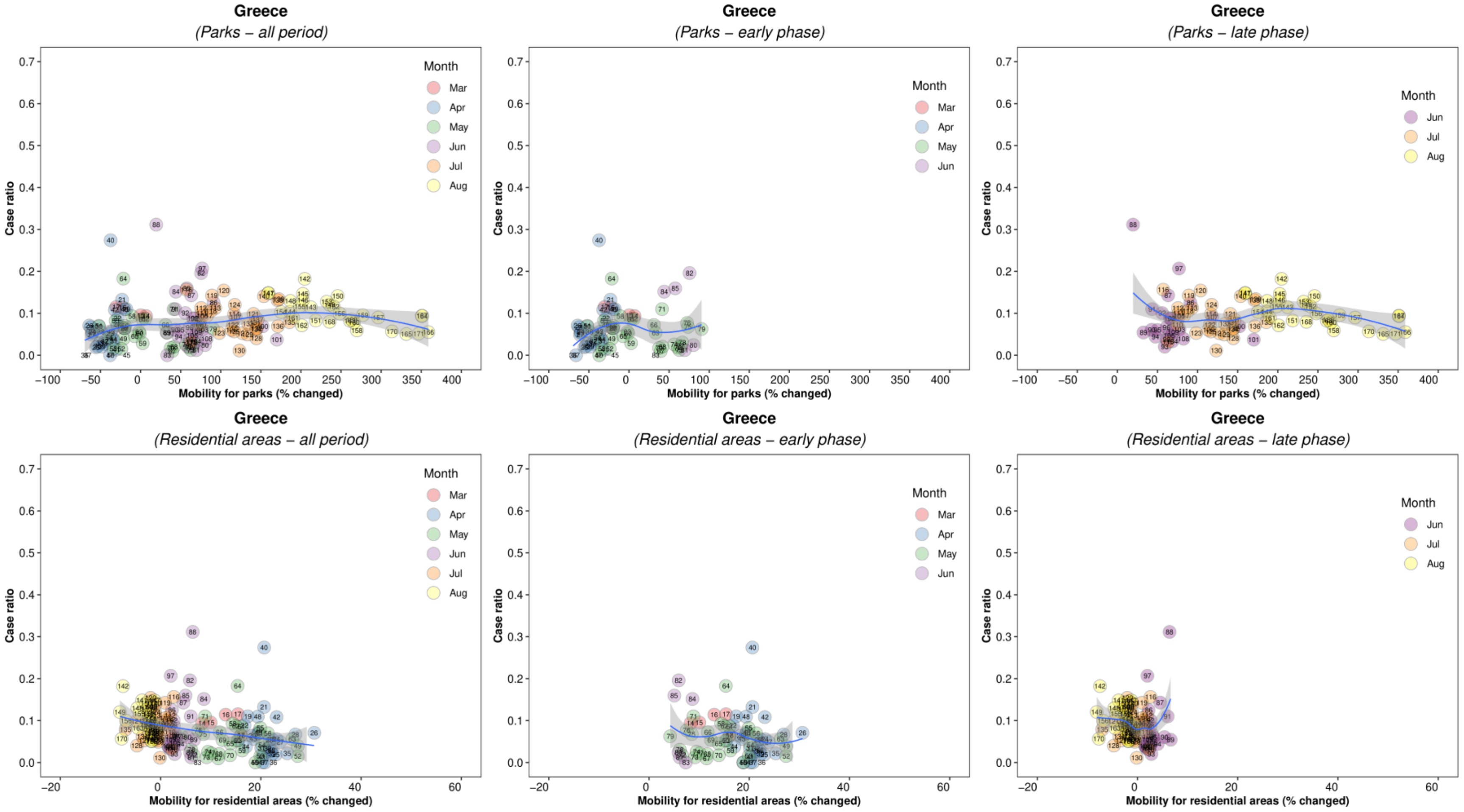
Greece
(Parks – all period)
Greece
(Parks – early phase)
Greece
(Parks – late phase)
Greece
(Residential areas – all period)
Greece
(Residential areas – early phase)
Greece
(Residential areas – late phase)
Case ratio
Mobility for parks (% changed)
Mobility for residential areas (% changed)
Month
Mar
Apr
May
Jun
Jul
Aug

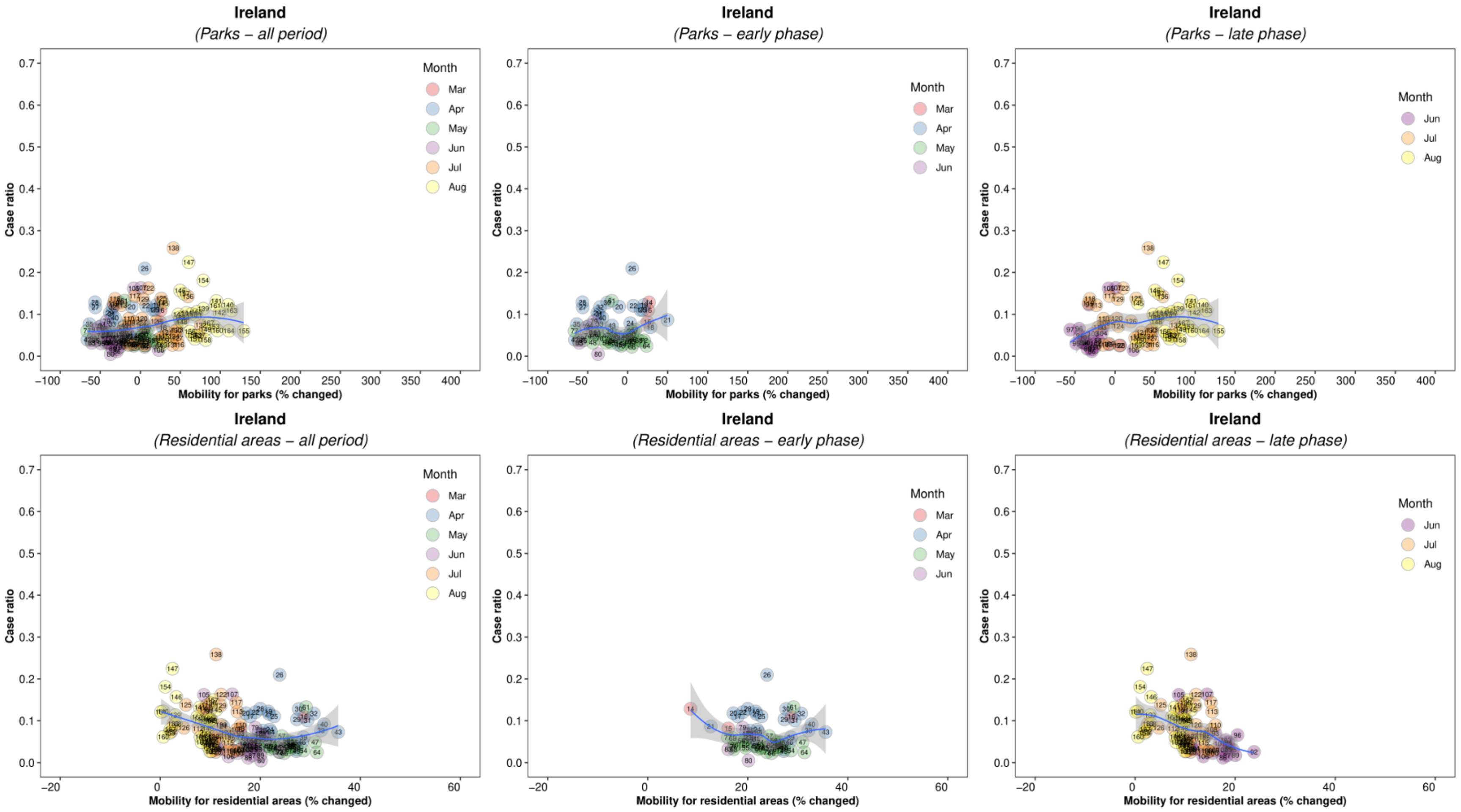
Ireland
(Parks – all period)
Ireland
(Parks – early phase)
Ireland
(Parks – late phase)
Ireland
(Residential areas – all period)
Ireland
(Residential areas – early phase)
Ireland
(Residential areas – late phase)
Case ratio
Mobility for parks (% changed)
Mobility for residential areas (% changed)
Month
Mar
Apr
May
Jun
Jul
Aug

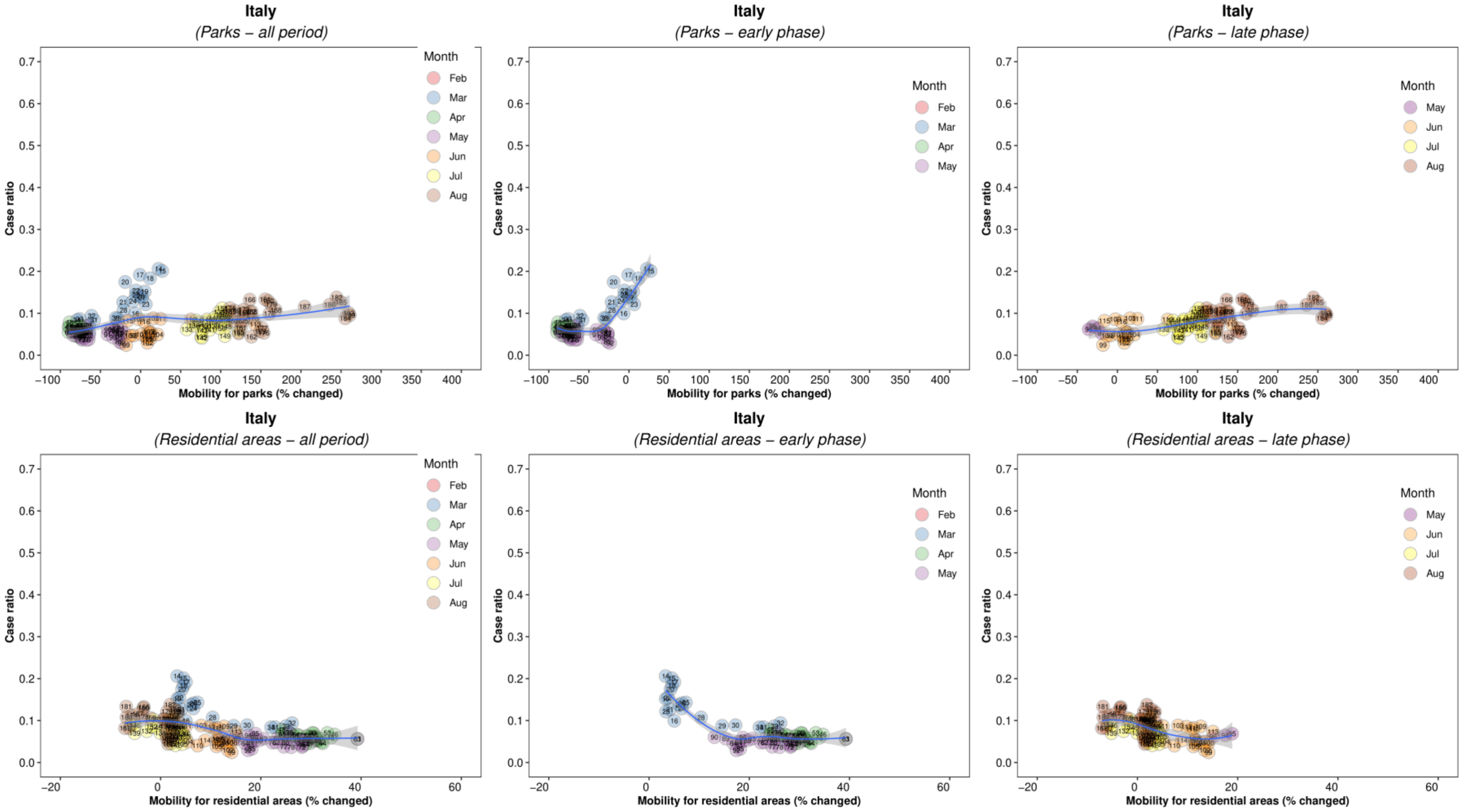
Italy
(Parks – all period)
Italy
(Parks – early phase)
Italy
(Parks – late phase)
Italy
(Residential areas – all period)
Italy
(Residential areas – early phase)
Italy
(Residential areas – late phase)
Case ratio
Mobility for parks (% changed)
Mobility for residential areas (% changed)
Month
Feb
Mar
Apr
May
Jun
Jul
Aug

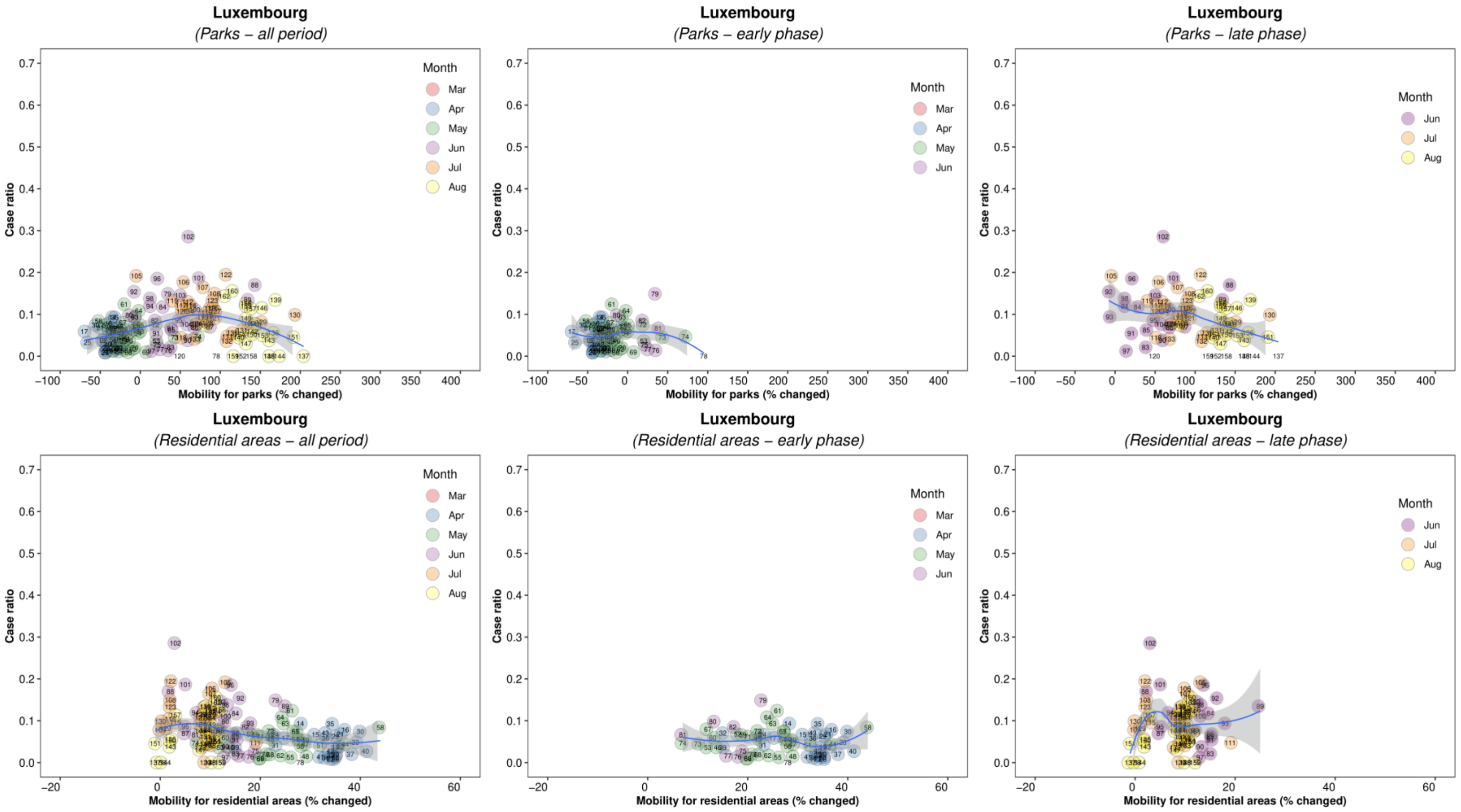
Luxembourg
(Parks – all period)
Luxembourg
(Parks – early phase)
Luxembourg
(Parks – late phase)
Luxembourg
(Residential areas – all period)
Luxembourg
(Residential areas – early phase)
Luxembourg
(Residential areas – late phase)
Case ratio
Mobility for parks (% changed)
Mobility for residential areas (% changed)
Month
Mar
Apr
May
Jun
Jul
Aug

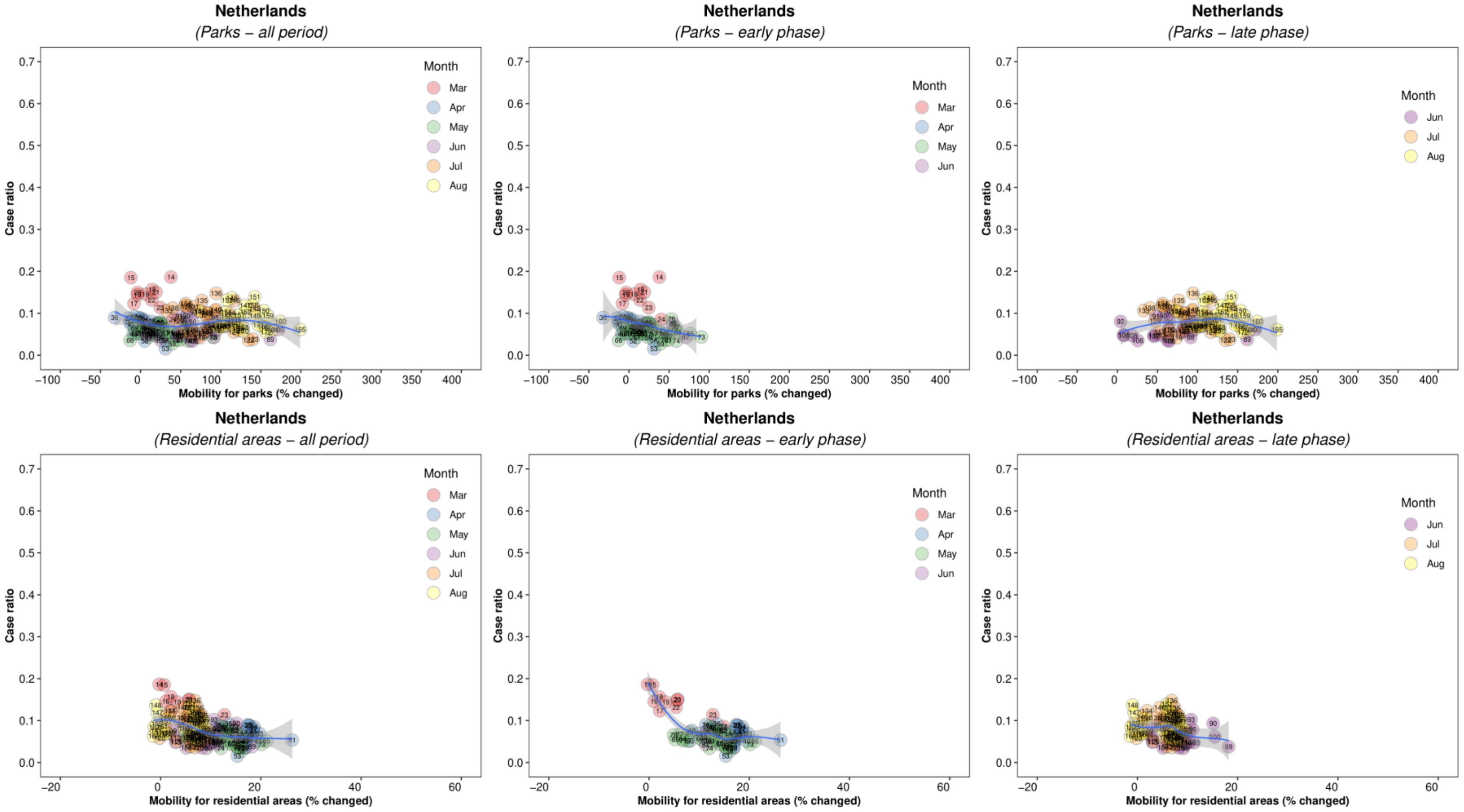
Netherlands
(Parks – all period)
Netherlands
(Parks – early phase)
Netherlands
(Parks – late phase)
Netherlands
(Residential areas – all period)
Netherlands
(Residential areas – early phase)
Netherlands
(Residential areas – late phase)
Case ratio
Mobility for parks (% changed)
Mobility for residential areas (% changed)
Month
Mar
Apr
May
Jun
Jul
Aug

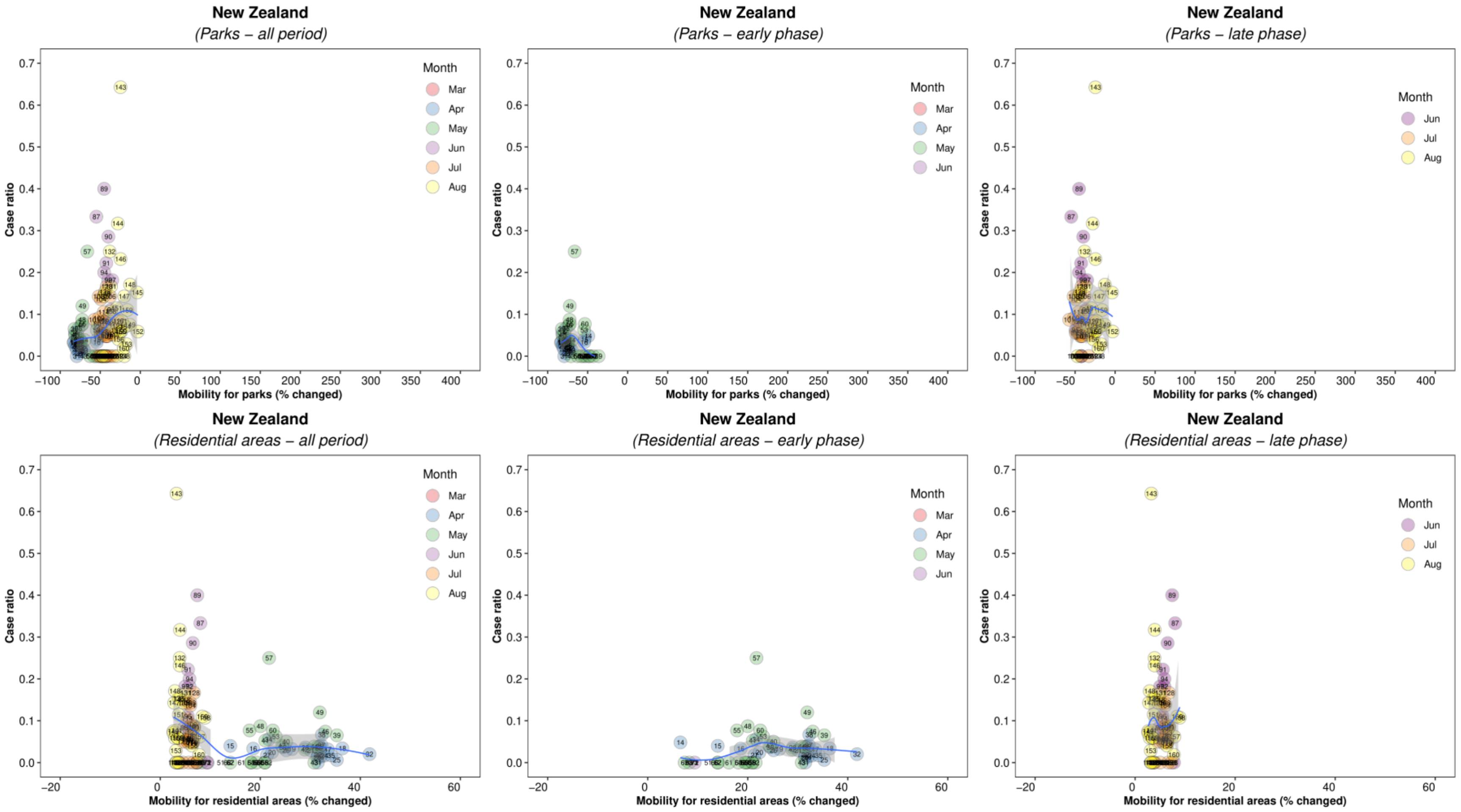
New Zealand
(Parks – all period)
Month
Mar
Apr
May
Jun
Jul
Aug
Case ratio
Mobility for parks (% changed)
New Zealand
(Parks – early phase)
Month
Mar
Apr
May
Jun
Case ratio
Mobility for parks (% changed)
New Zealand
(Parks – late phase)
Month
Jun
Jul
Aug
Case ratio
Mobility for parks (% changed)
New Zealand
(Residential areas – all period)
Month
Mar
Apr
May
Jun
Jul
Aug
Case ratio
Mobility for residential areas (% changed)
New Zealand
(Residential areas – early phase)
Month
Mar
Apr
May
Jun
Case ratio
Mobility for residential areas (% changed)
New Zealand
(Residential areas – late phase)
Month
Jun
Jul
Aug
Case ratio
Mobility for residential areas (% changed)

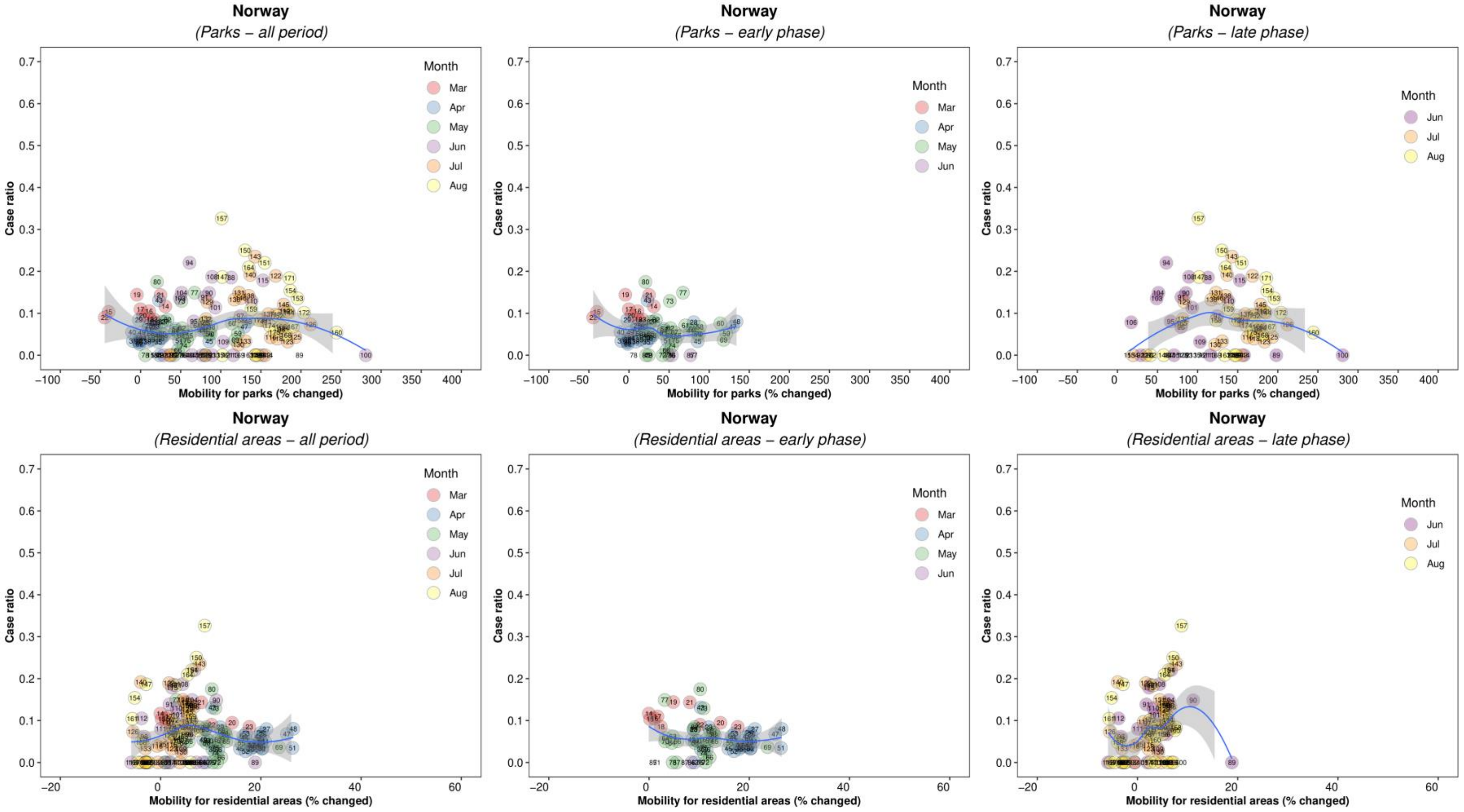
Norway
(Parks – all period)
Norway
(Parks – early phase)
Norway
(Parks – late phase)
Norway
(Residential areas – all period)
Norway
(Residential areas – early phase)
Norway
(Residential areas – late phase)
Case ratio
Mobility for parks (% changed)
Mobility for residential areas (% changed)
Month
Mar
Apr
May
Jun
Jul
Aug

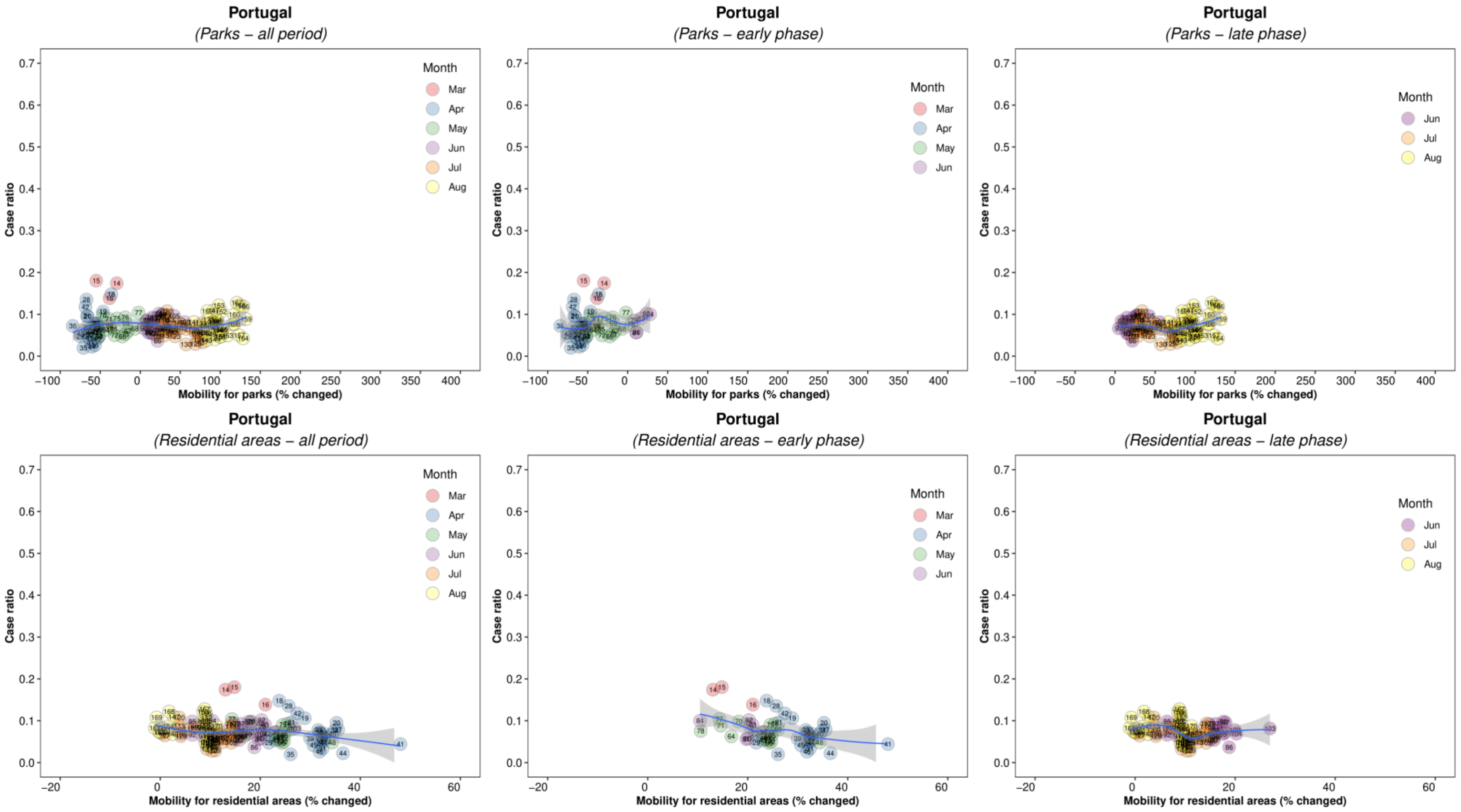
Portugal
(Parks – all period)
Portugal
(Parks – early phase)
Portugal
(Parks – late phase)
Portugal
(Residential areas – all period)
Portugal
(Residential areas – early phase)
Portugal
(Residential areas – late phase)
Case ratio
Mobility for parks (% changed)
Mobility for residential areas (% changed)
Month
Mar
Apr
May
Jun
Jul
Aug

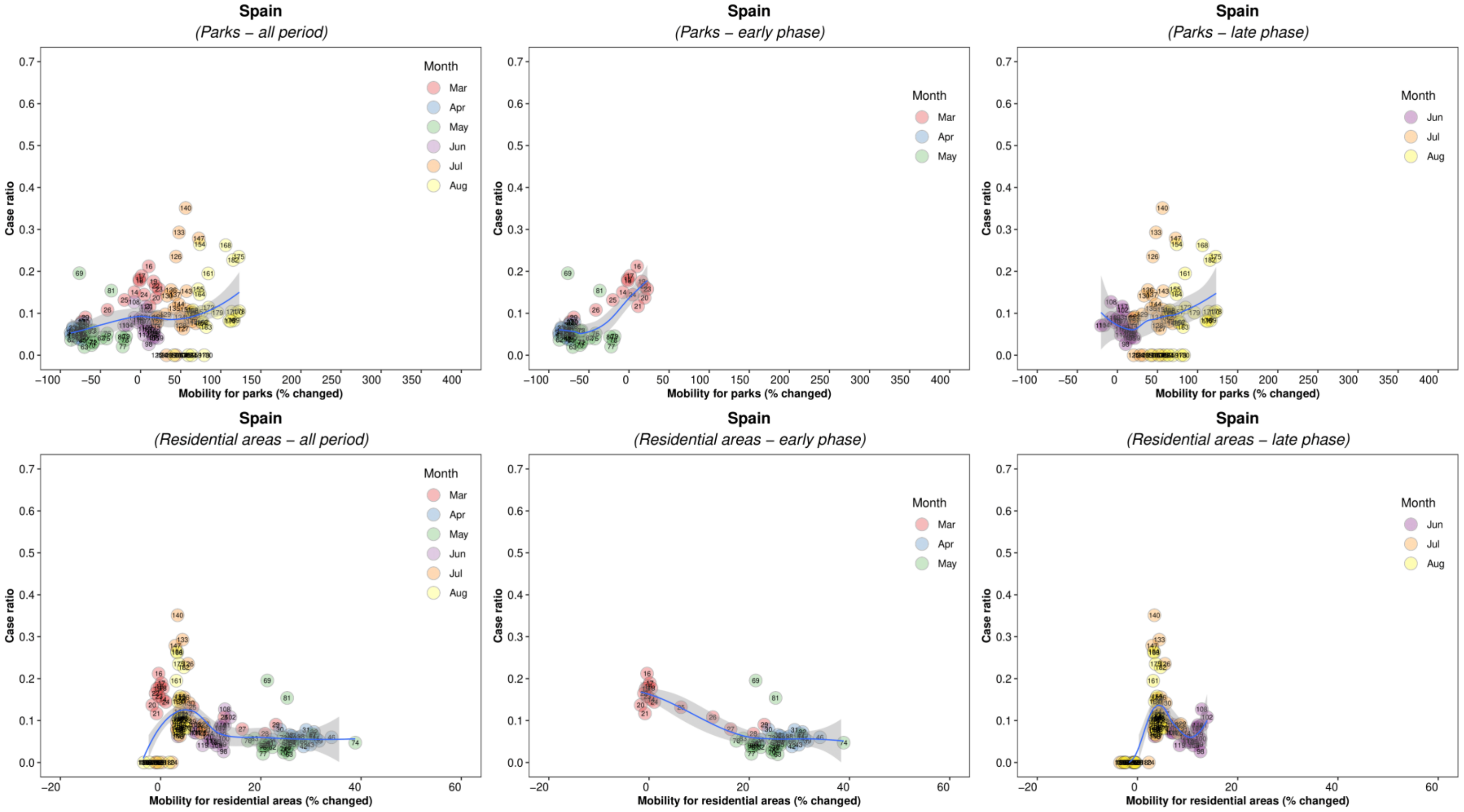
Spain
(Parks – all period)
Spain
(Parks – early phase)
Spain
(Parks – late phase)
Spain
(Residential areas – all period)
Spain
(Residential areas – early phase)
Spain
(Residential areas – late phase)
Case ratio
Mobility for parks (% changed)
Mobility for residential areas (% changed)
Month
Mar
Apr
May
Jun
Jul
Aug

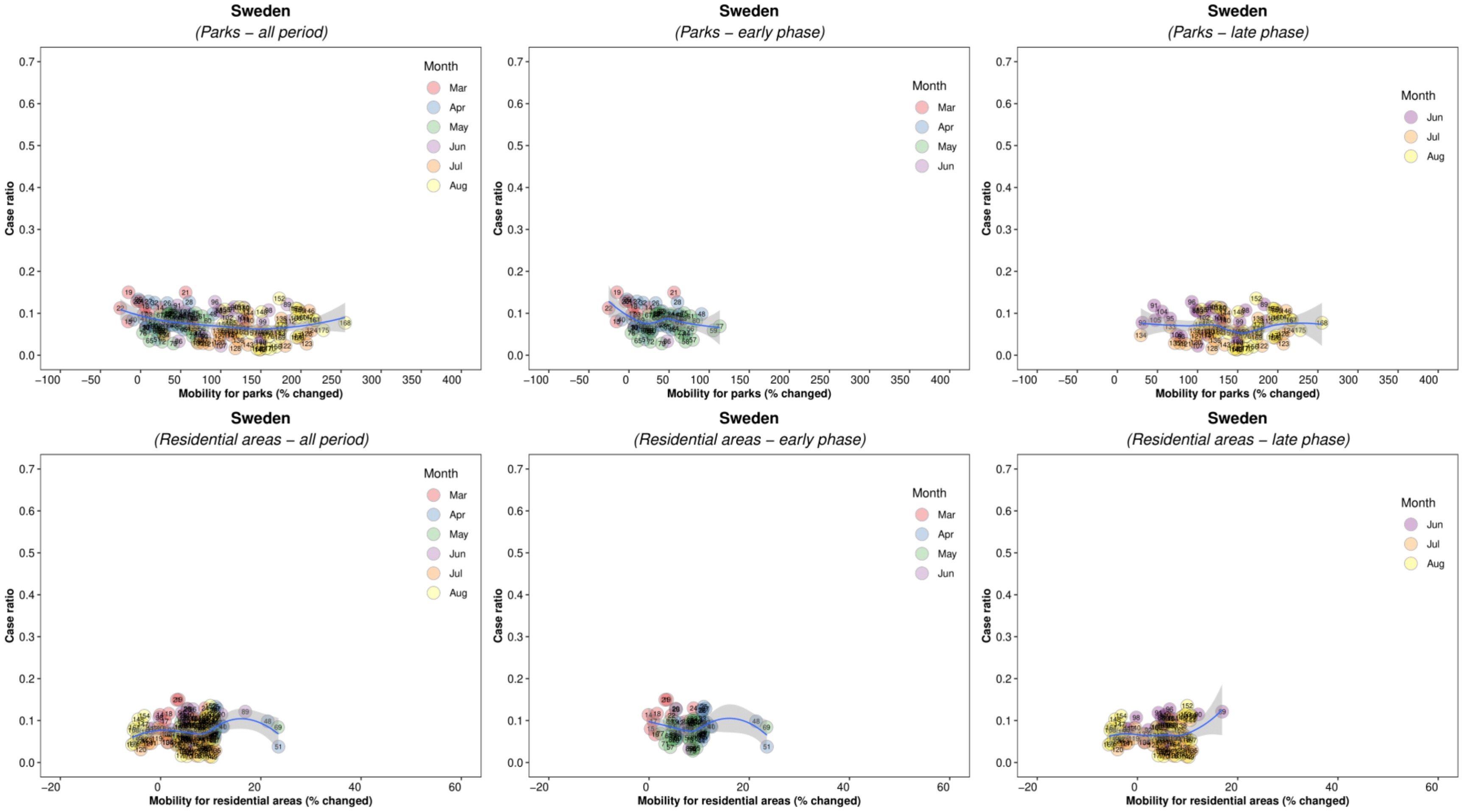
Sweden
(Parks – all period)
Sweden
(Parks – early phase)
Sweden
(Parks – late phase)
Sweden
(Residential areas – all period)
Sweden
(Residential areas – early phase)
Sweden
(Residential areas – late phase)
Case ratio
Mobility for parks (% changed)
Mobility for residential areas (% changed)
Month
Mar
Apr
May
Jun
Jul
Aug

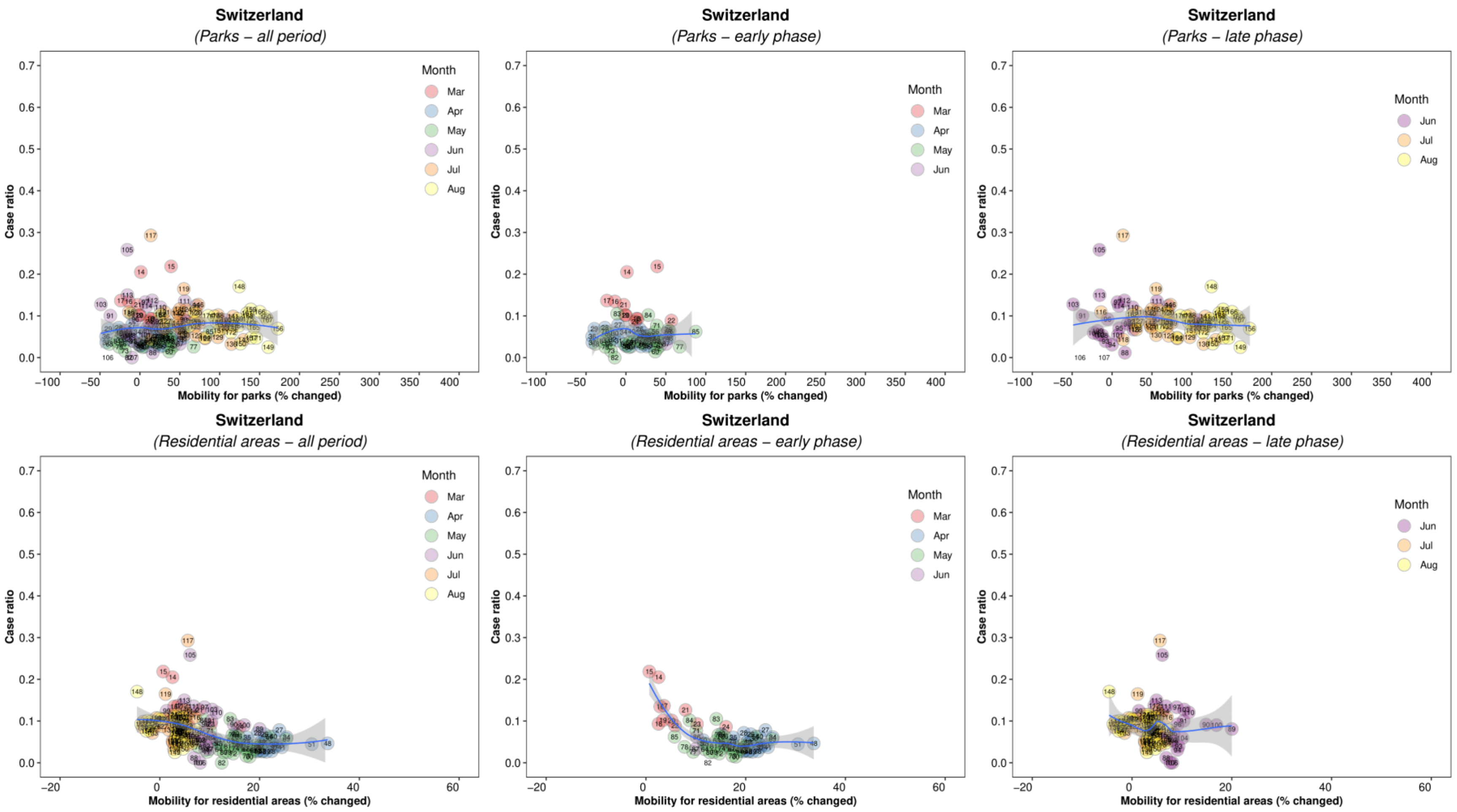
Switzerland
(Parks – all period)
Switzerland
(Parks – early phase)
Switzerland
(Parks – late phase)
Switzerland
(Residential areas – all period)
Switzerland
(Residential areas – early phase)
Switzerland
(Residential areas – late phase)
Case ratio
Mobility for parks (% changed)
Mobility for residential areas (% changed)
Month
Mar
Apr
May
Jun
Jul
Aug

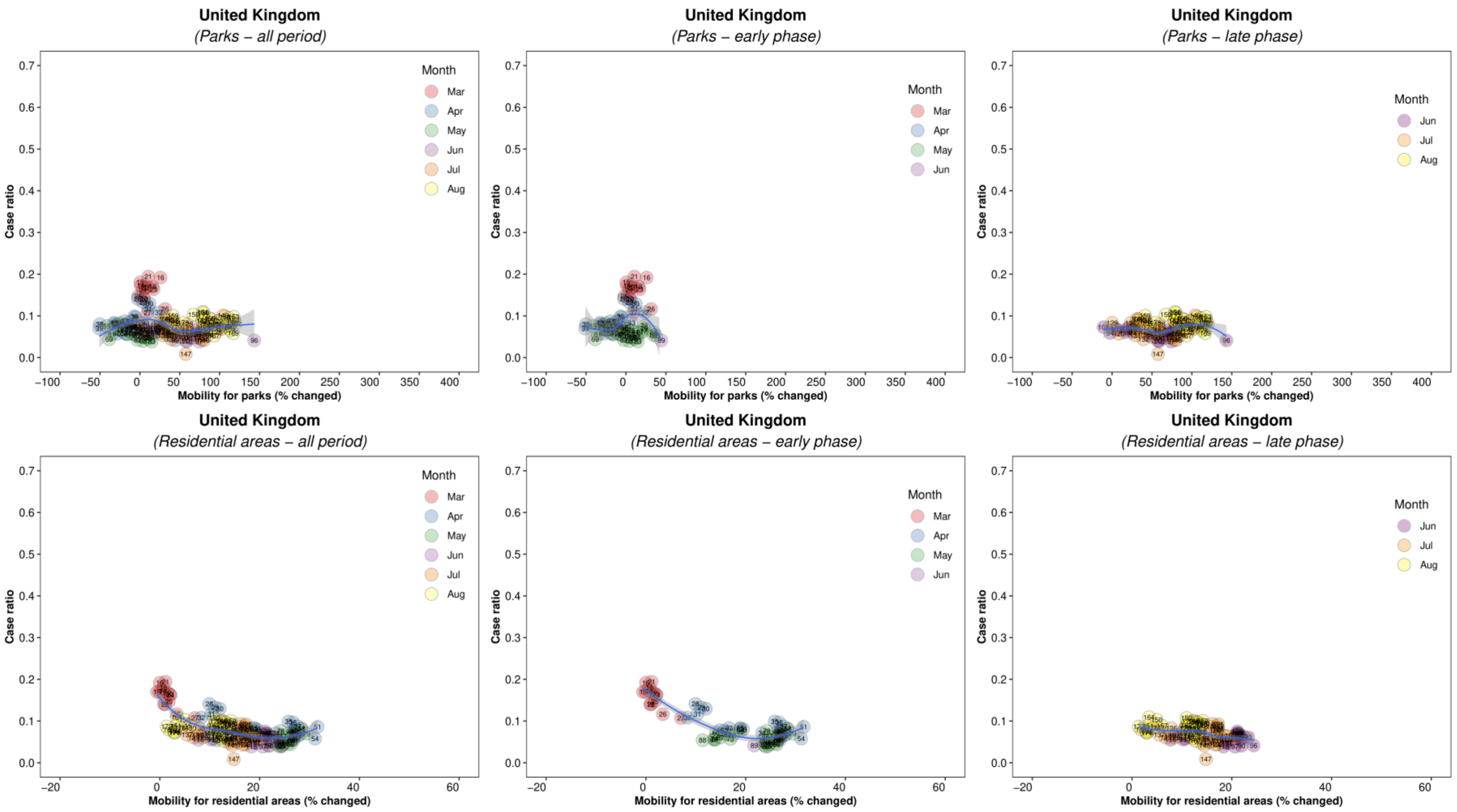
United Kingdom
(Parks – all period)
United Kingdom
(Parks – early phase)
United Kingdom
(Parks – late phase)
Mobility for parks (% changed)
United Kingdom
(Residential areas – all period)
United Kingdom
(Residential areas – early phase)
United Kingdom
(Residential areas – late phase)
Mobility for residential areas (% changed)
Case ratio
Month
Mar
Apr
May
Jun
Jul
Aug

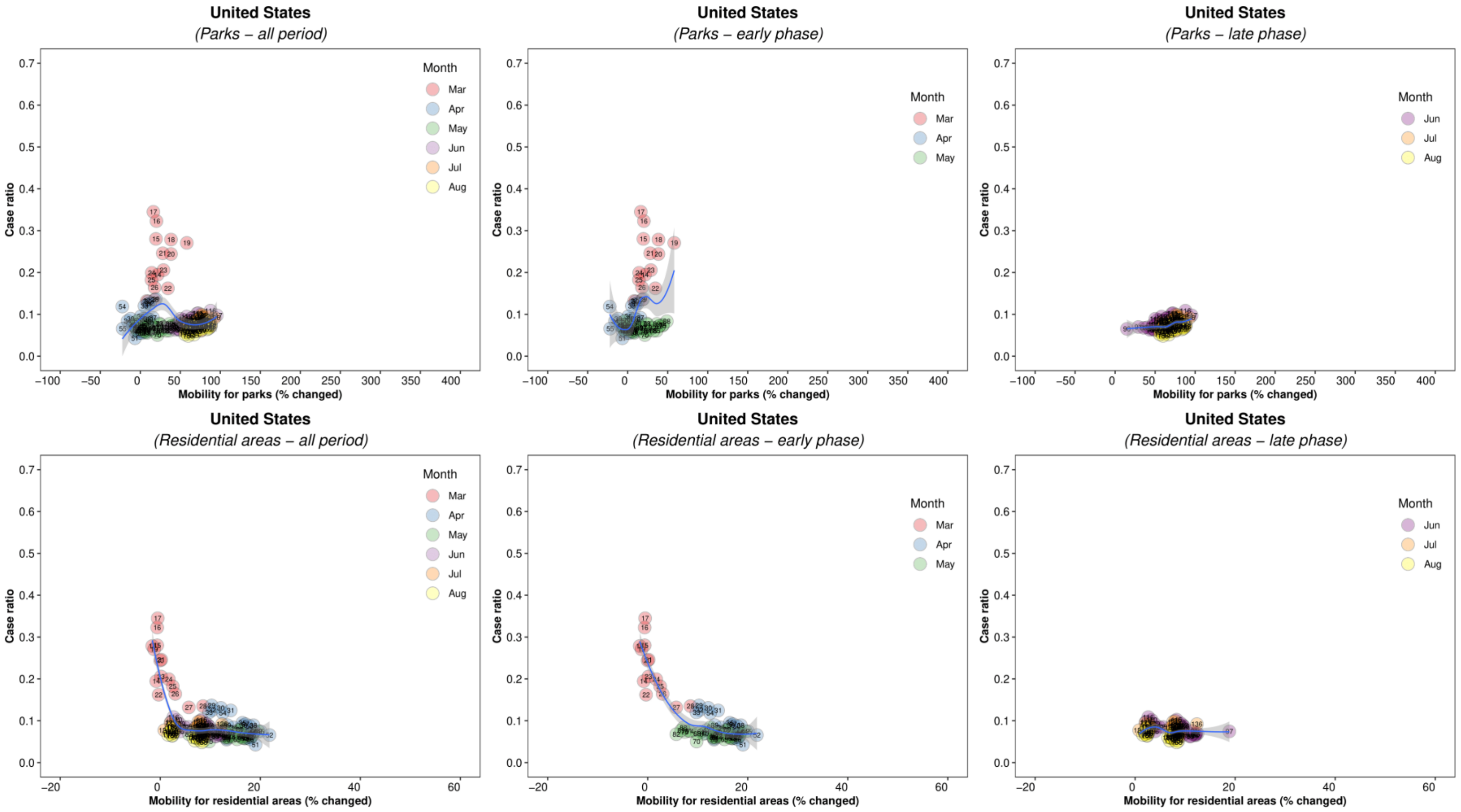
United States
(Parks – all period)
United States
(Parks – early phase)
United States
(Parks – late phase)
Month
Mar
Apr
May
Jun
Jul
Aug
Case ratio
Mobility for parks (% changed)
United States
(Residential areas – all period)
United States
(Residential areas – early phase)
United States
(Residential areas – late phase)
Mobility for residential areas (% changed)

Supplementary Figure 2B. Association between new daily COVID-19 case ratio and mobility changes in 36 countries by early and late phase and other places (parks and residential areas) visited. Asia, Eastern Europe, Latin America, and Caribbean.

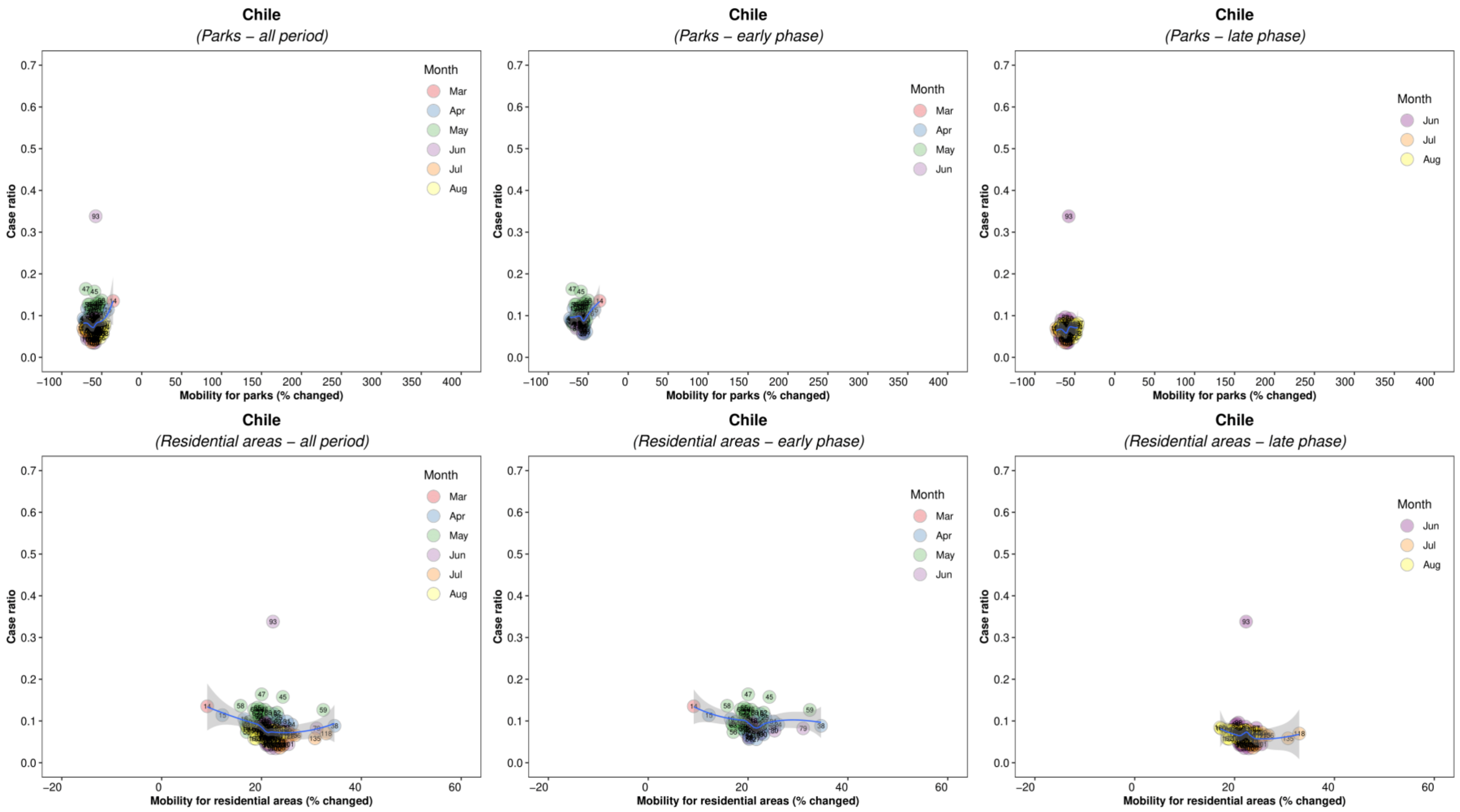

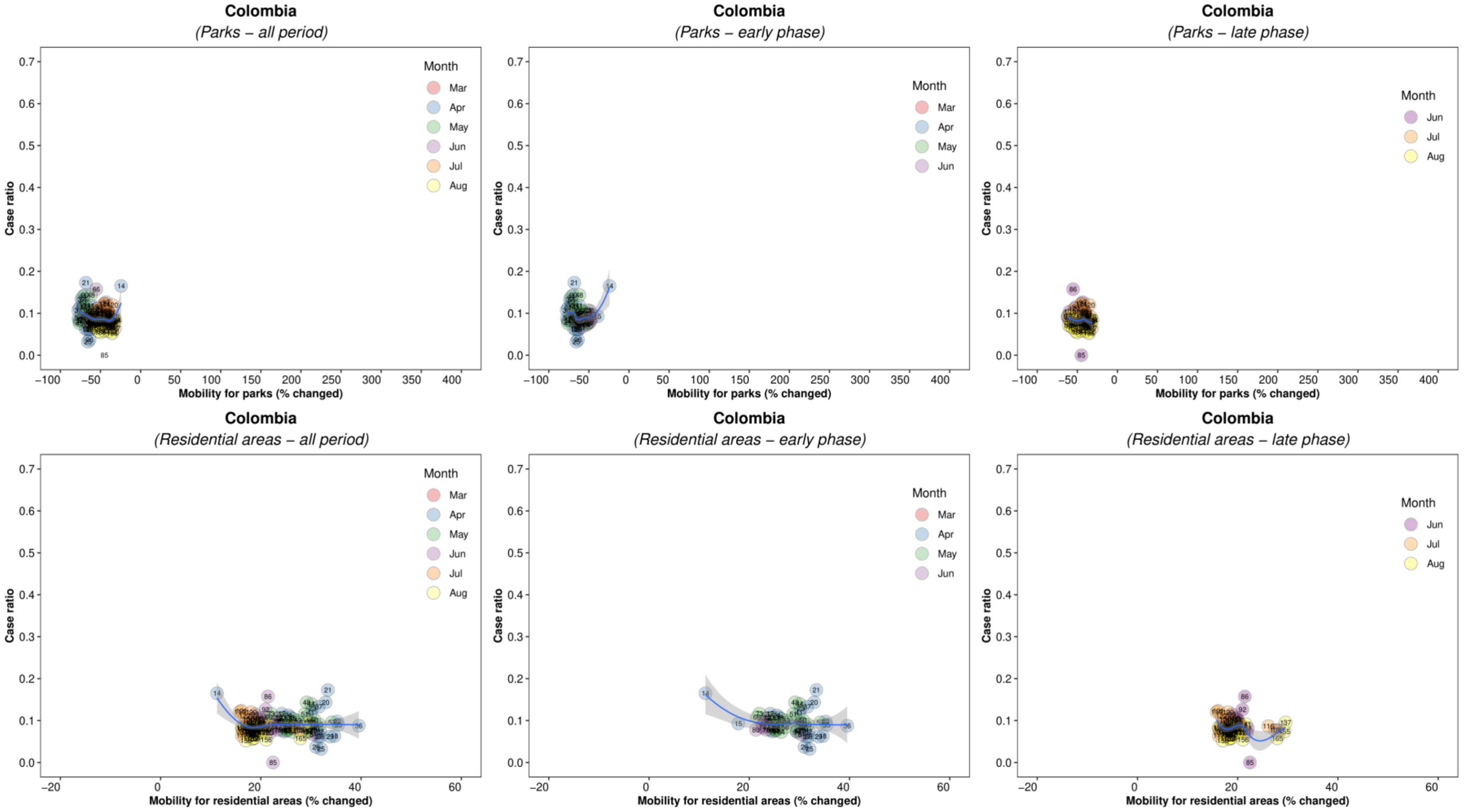
Colombia
(Parks – all period)
Colombia
(Parks – early phase)
Colombia
(Parks – late phase)
Colombia
(Residential areas – all period)
Colombia
(Residential areas – early phase)
Colombia
(Residential areas – late phase)
Case ratio
Mobility for parks (% changed)
Mobility for residential areas (% changed)
Month
Mar
Apr
May
Jun
Jul
Aug

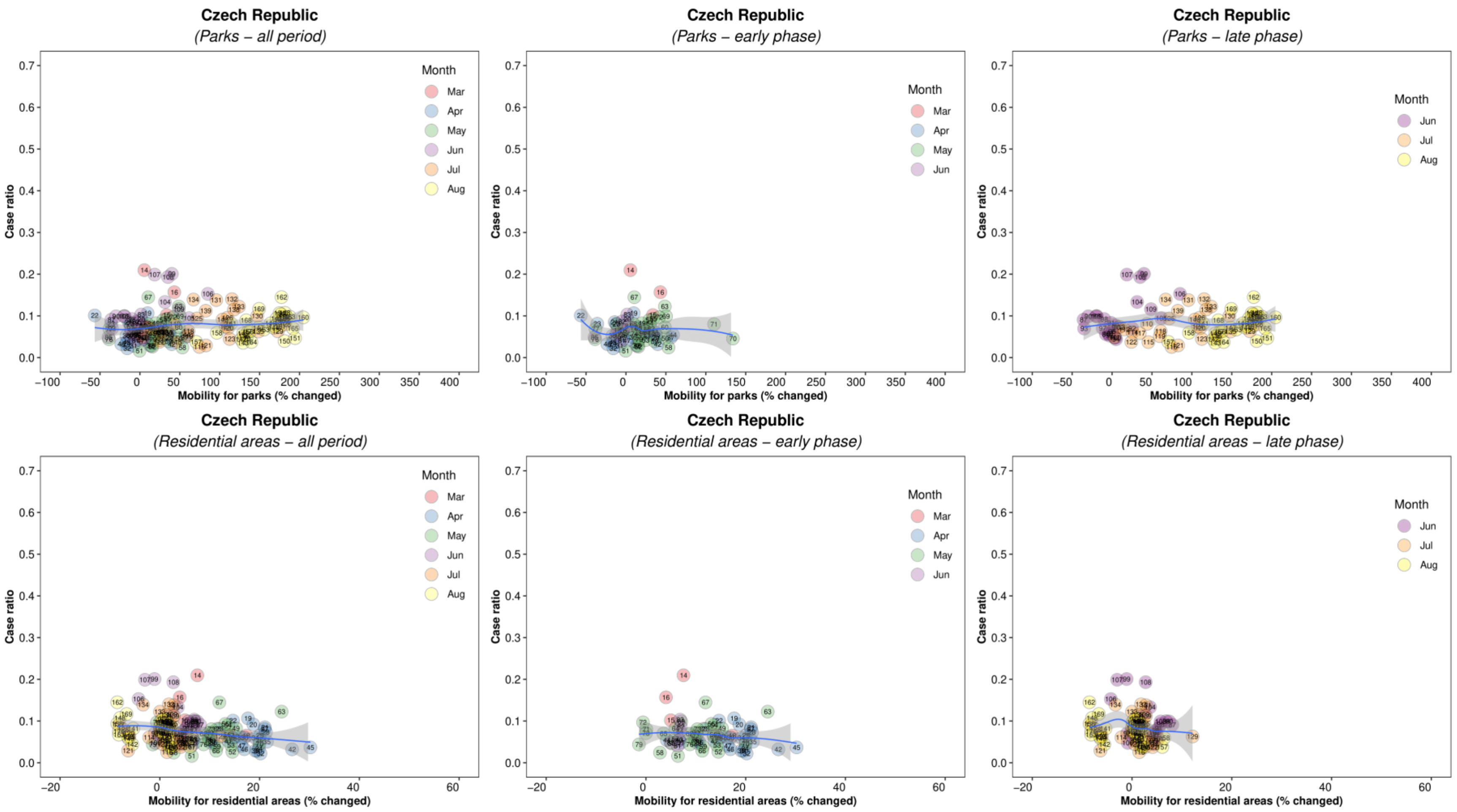
Czech Republic
(Parks – all period)
Czech Republic
(Parks – early phase)
Czech Republic
(Parks – late phase)
Case ratio
Mobility for parks (% changed)
Month
Mar
Apr
May
Jun
Jul
Aug
Czech Republic
(Residential areas – all period)
Czech Republic
(Residential areas – early phase)
Czech Republic
(Residential areas – late phase)
Mobility for residential areas (% changed)

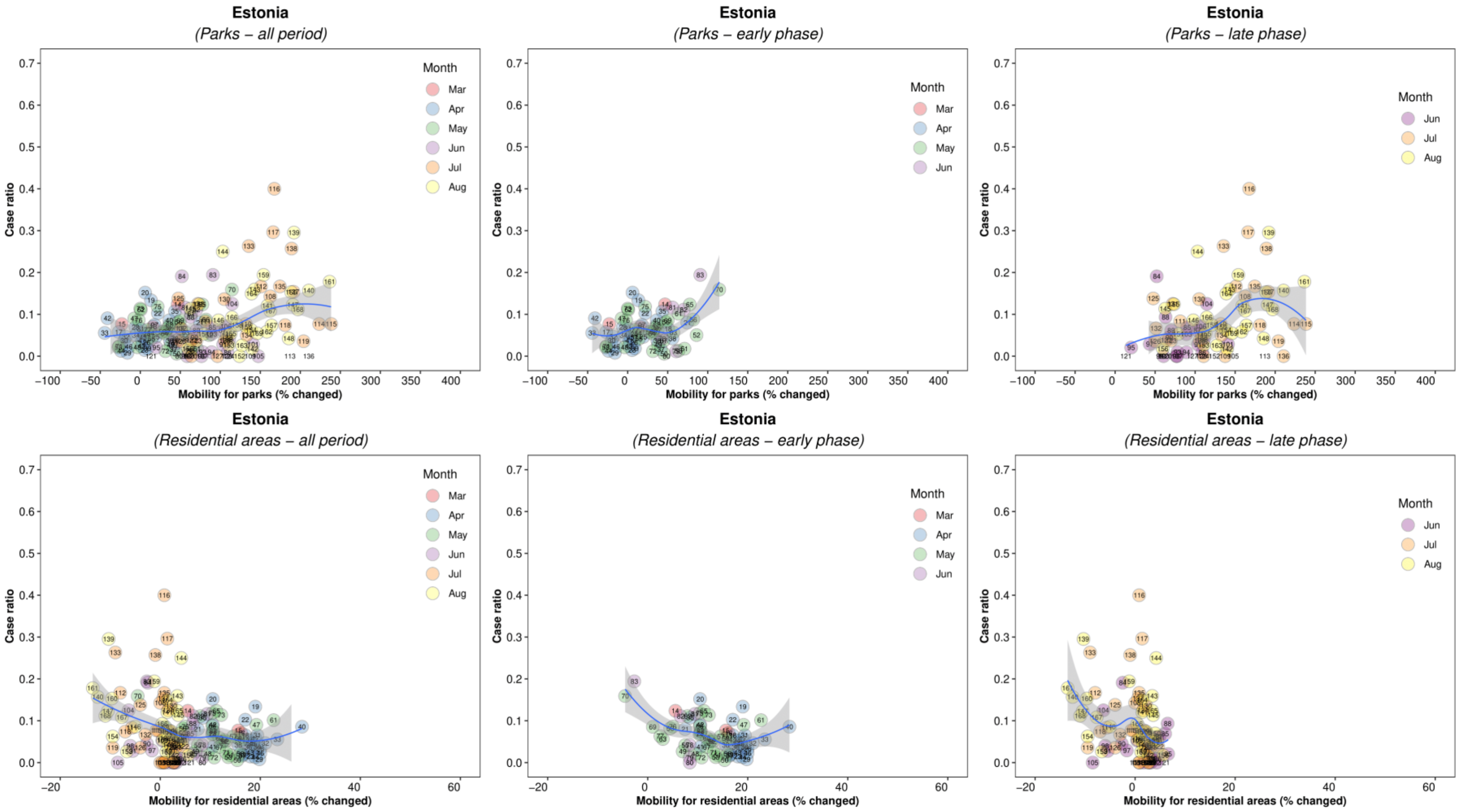
Estonia
(Parks – all period)
Estonia
(Parks – early phase)
Estonia
(Parks – late phase)
Estonia
(Residential areas – all period)
Estonia
(Residential areas – early phase)
Estonia
(Residential areas – late phase)
Case ratio
Mobility for parks (% changed)
Mobility for residential areas (% changed)
Month
Mar
Apr
May
Jun
Jul
Aug

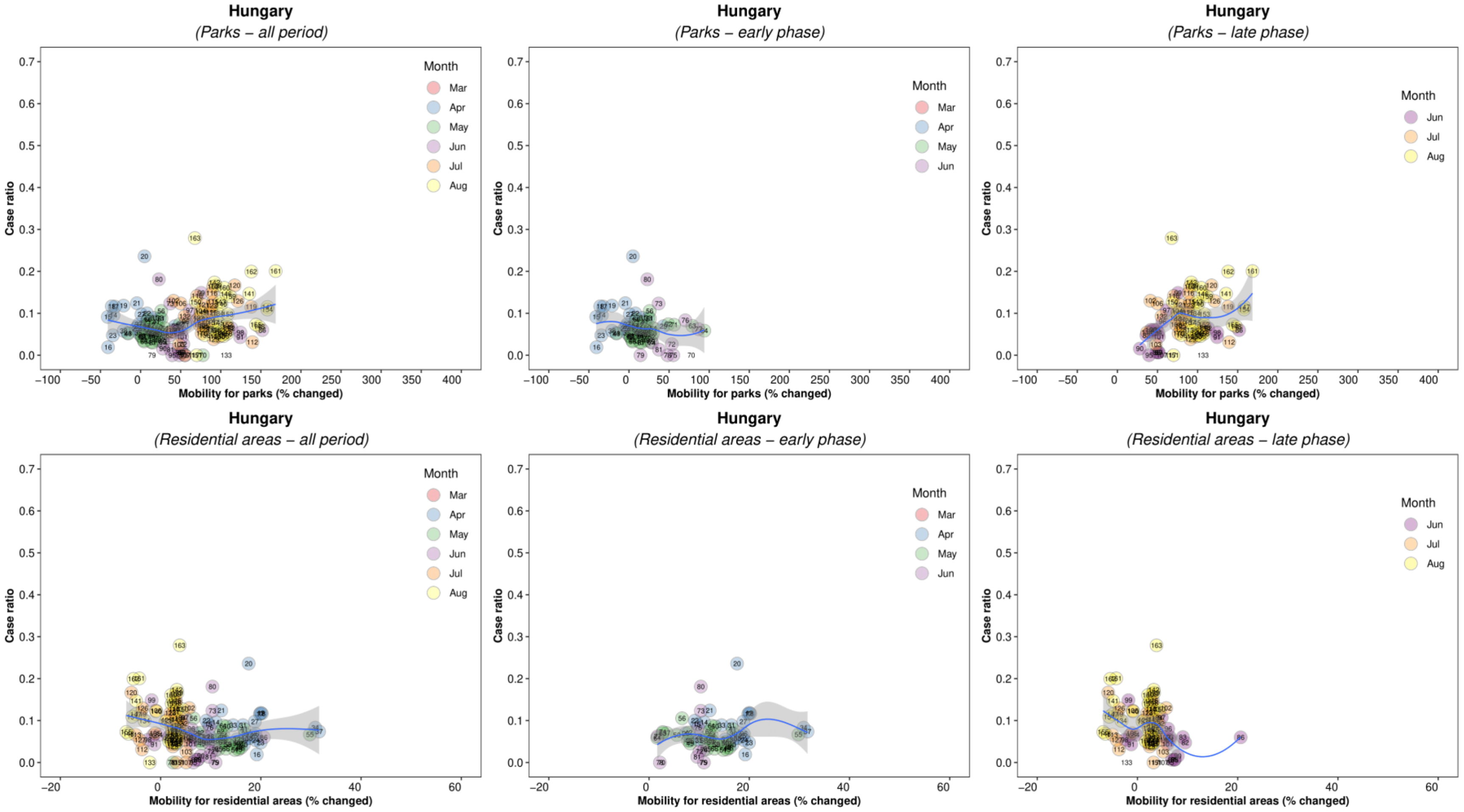
Hungary
(Parks – all period)
Hungary
(Parks – early phase)
Hungary
(Parks – late phase)
Hungary
(Residential areas – all period)
Hungary
(Residential areas – early phase)
Hungary
(Residential areas – late phase)
Case ratio
Mobility for parks (% changed)
Mobility for residential areas (% changed)
Month
Mar
Apr
May
Jun
Jul
Aug

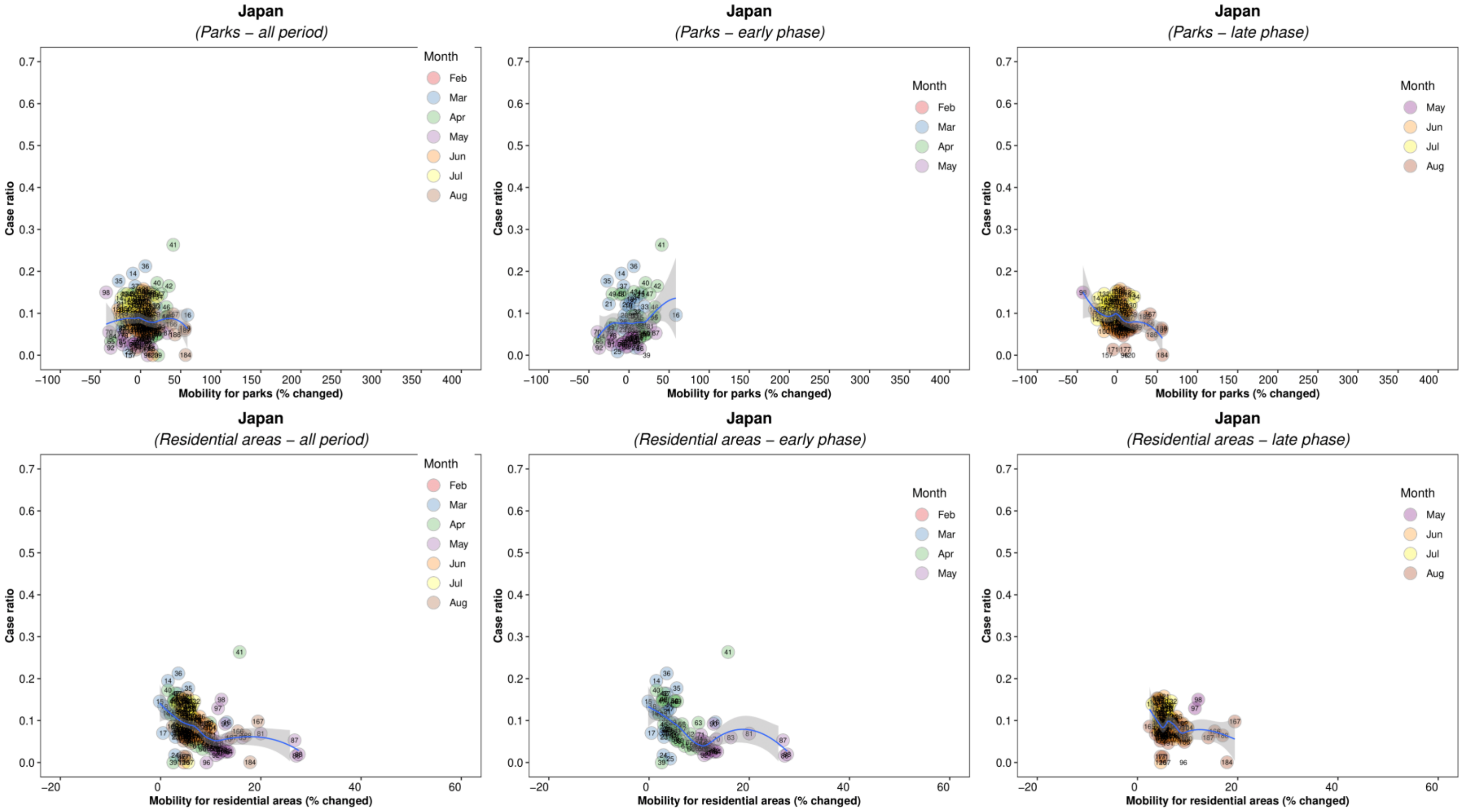
Japan
(Parks – all period)
Month
Feb
Mar
Apr
May
Jun
Jul
Aug
Case ratio
Mobility for parks (% changed)
Japan
(Parks – early phase)
Month
Feb
Mar
Apr
May
Case ratio
Mobility for parks (% changed)
Japan
(Parks – late phase)
Month
May
Jun
Jul
Aug
Case ratio
Mobility for parks (% changed)
Japan
(Residential areas – all period)
Month
Feb
Mar
Apr
May
Jun
Jul
Aug
Case ratio
Mobility for residential areas (% changed)
Japan
(Residential areas – early phase)
Month
Feb
Mar
Apr
May
Case ratio
Mobility for residential areas (% changed)
Japan
(Residential areas – late phase)
Month
May
Jun
Jul
Aug
Case ratio
Mobility for residential areas (% changed)

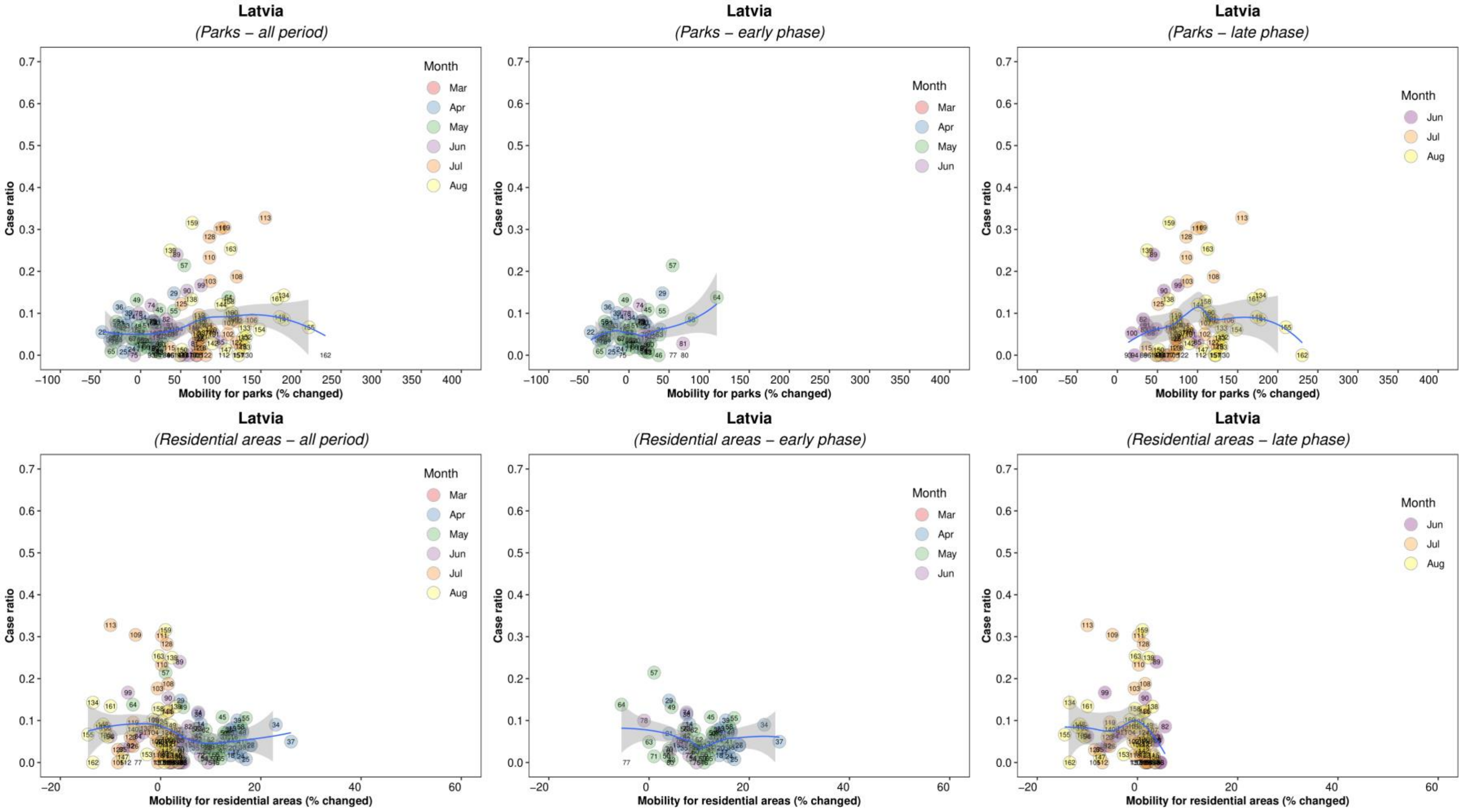

Latvia
(Parks – all period)
Latvia
(Parks – early phase)
Latvia
(Parks – late phase)
Latvia
(Residential areas – all period)
Latvia
(Residential areas – early phase)
Latvia
(Residential areas – late phase)
Case ratio
Mobility for parks (% changed)
Mobility for residential areas (% changed)
Month
Mar
Apr
May
Jun
Jul
Aug

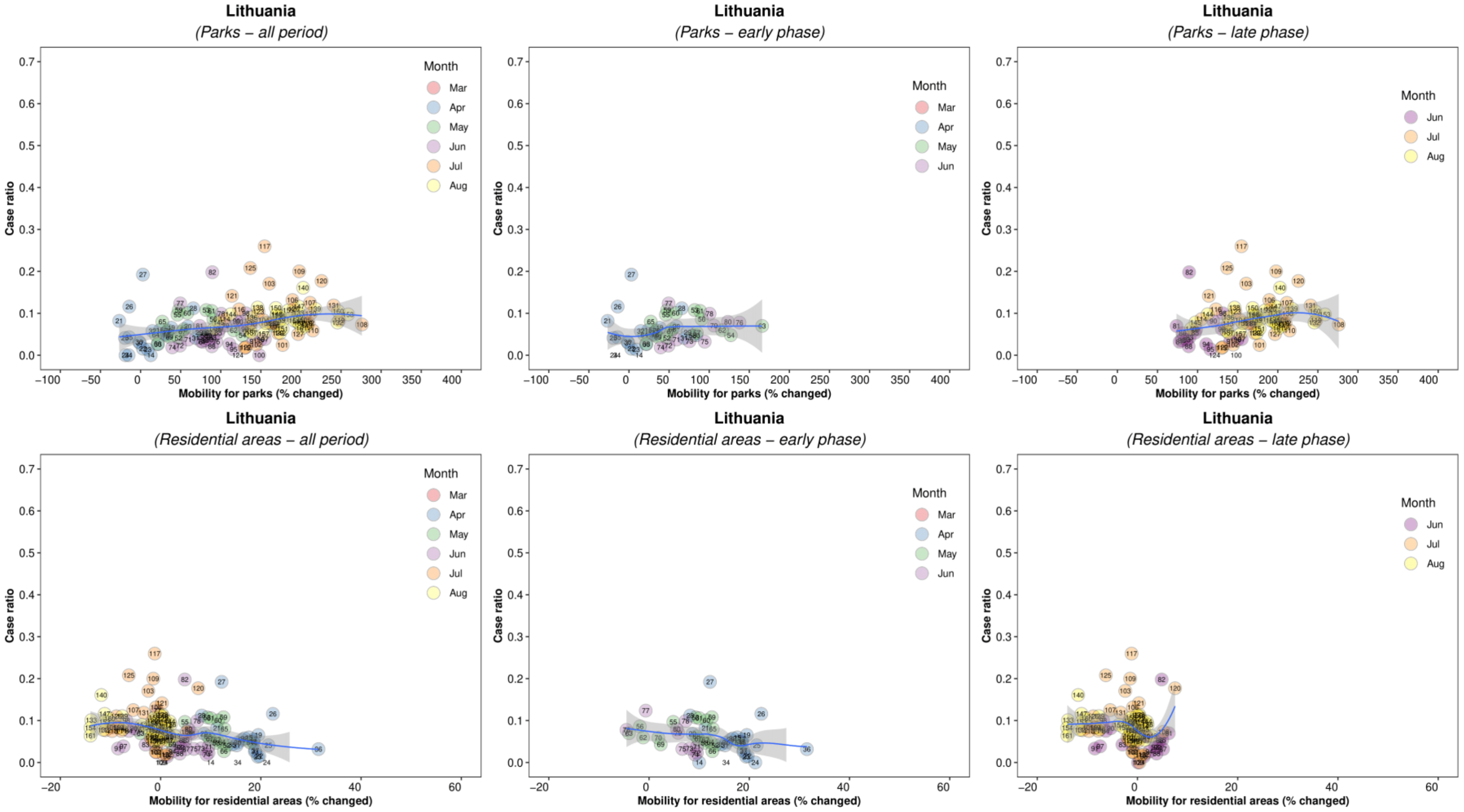
Lithuania
(Parks – all period)
Lithuania
(Parks – early phase)
Lithuania
(Parks – late phase)
Lithuania
(Residential areas – all period)
Lithuania
(Residential areas – early phase)
Lithuania
(Residential areas – late phase)
Case ratio
Mobility for parks (% changed)
Mobility for residential areas (% changed)
Month
Mar
Apr
May
Jun
Jul
Aug

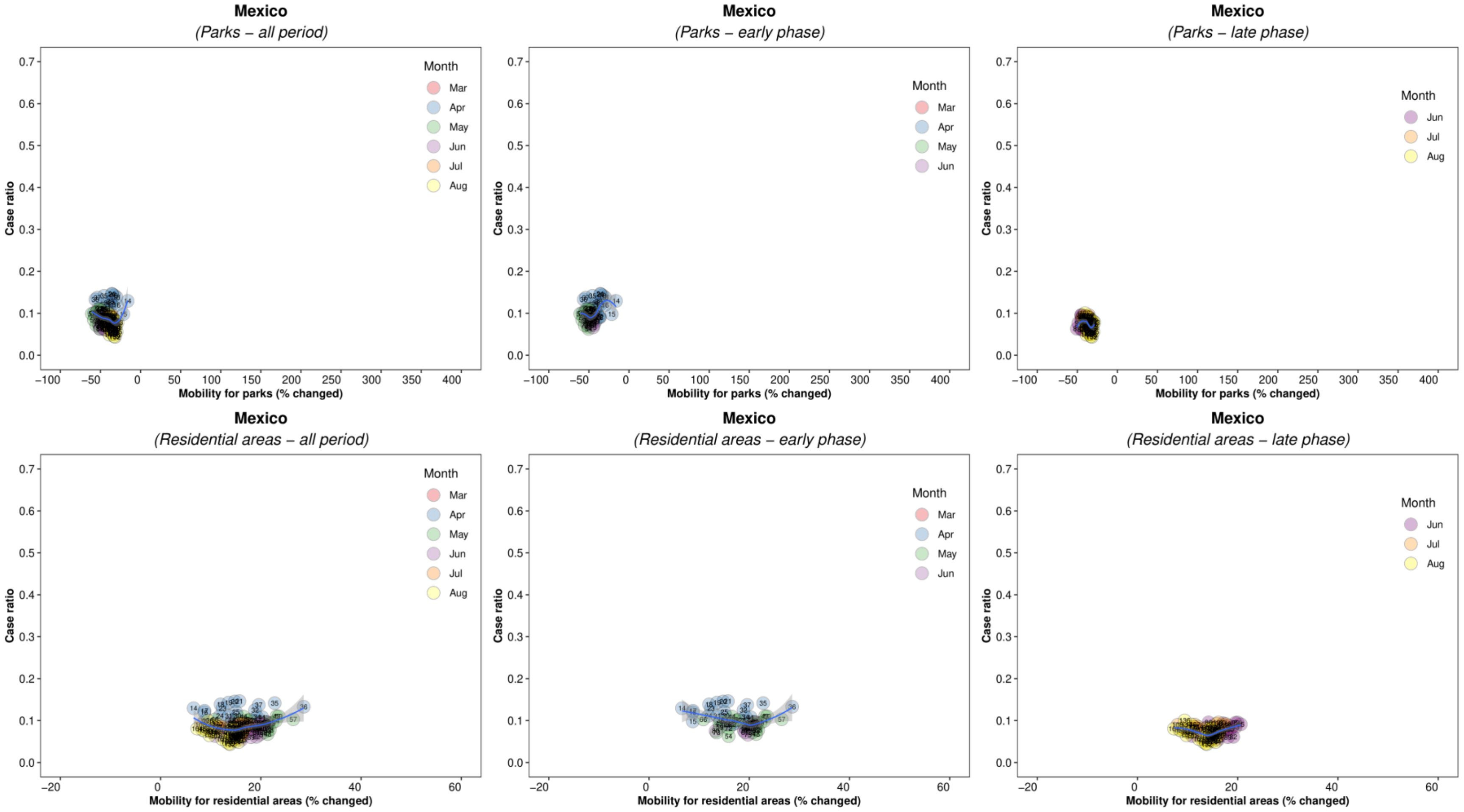
Mexico
(Parks – all period)
Mexico
(Parks – early phase)
Mexico
(Parks – late phase)
Mexico
(Residential areas – all period)
Mexico
(Residential areas – early phase)
Mexico
(Residential areas – late phase)
Case ratio
Mobility for parks (% changed)
Mobility for residential areas (% changed)
Month
Mar
Apr
May
Jun
Jul
Aug

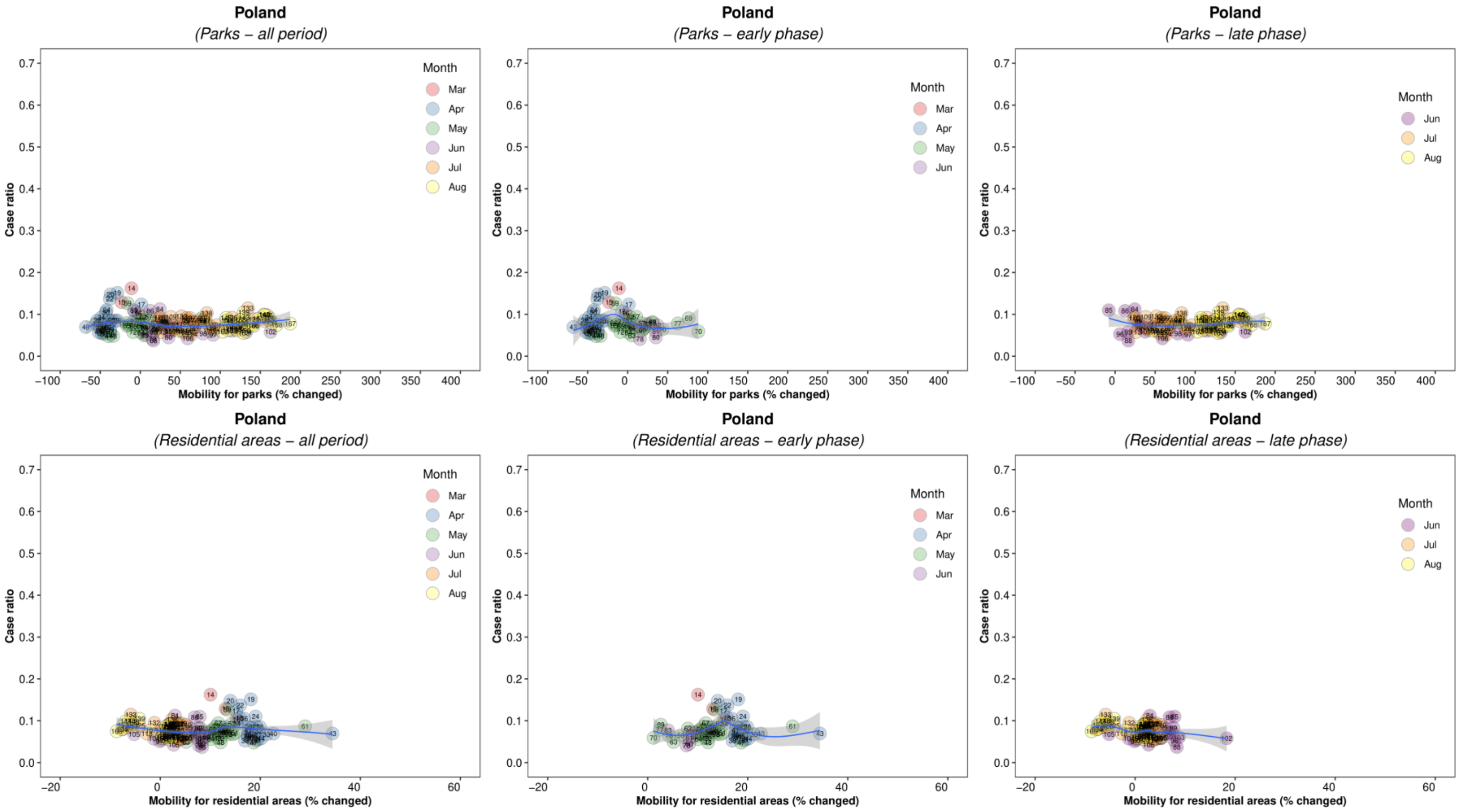
Poland
(Parks – all period)
Poland
(Parks – early phase)
Poland
(Parks – late phase)
Case ratio
Mobility for parks (% changed)
Month
Mar
Apr
May
Jun
Jul
Aug
Poland
(Residential areas – all period)
Poland
(Residential areas – early phase)
Poland
(Residential areas – late phase)
Mobility for residential areas (% changed)

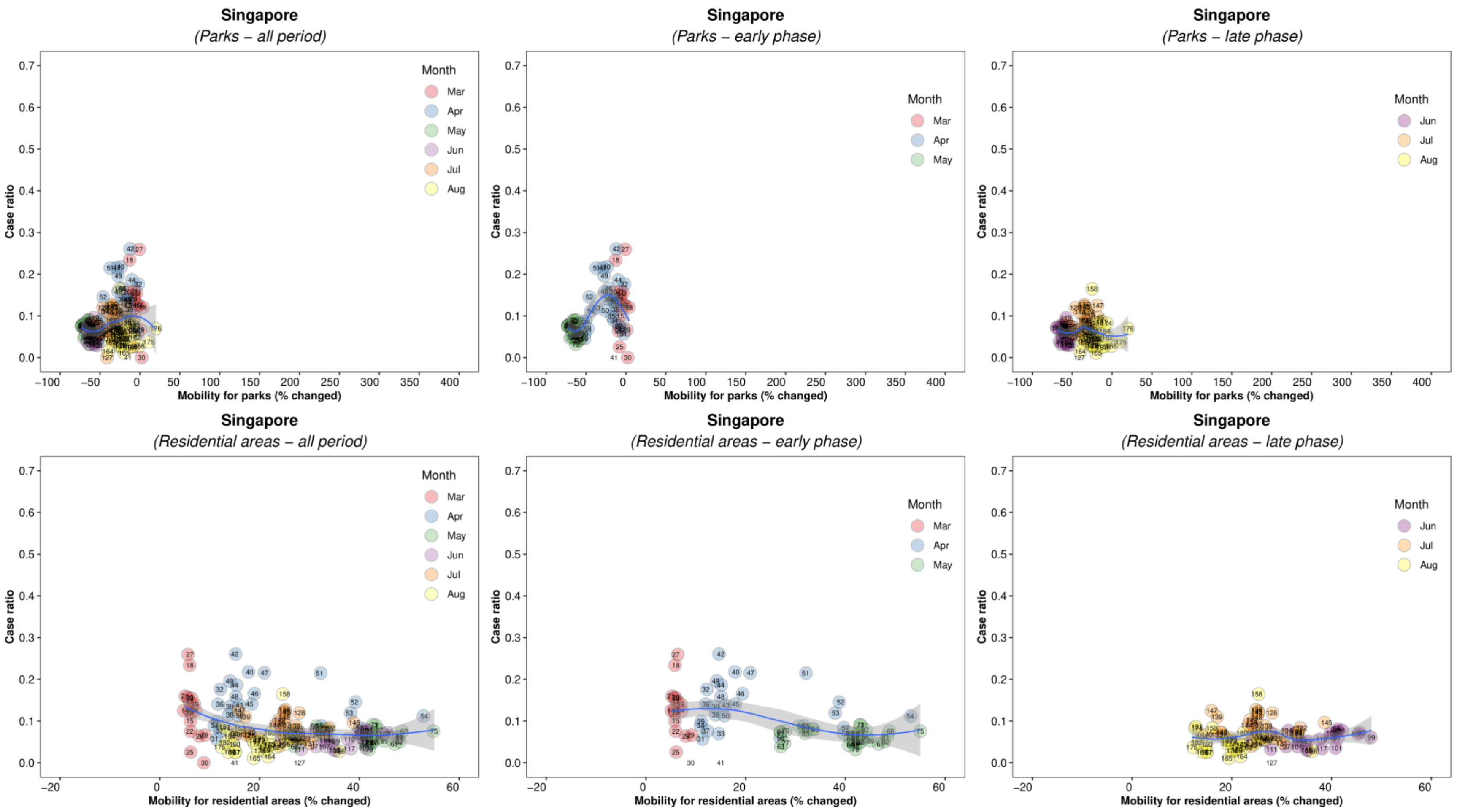
Singapore
(Parks – all period)
Singapore
(Parks – early phase)
Singapore
(Parks – late phase)
Singapore
(Residential areas – all period)
Singapore
(Residential areas – early phase)
Singapore
(Residential areas – late phase)
Case ratio
Mobility for parks (% changed)
Mobility for residential areas (% changed)
Month
Mar
Apr
May
Jun
Jul
Aug

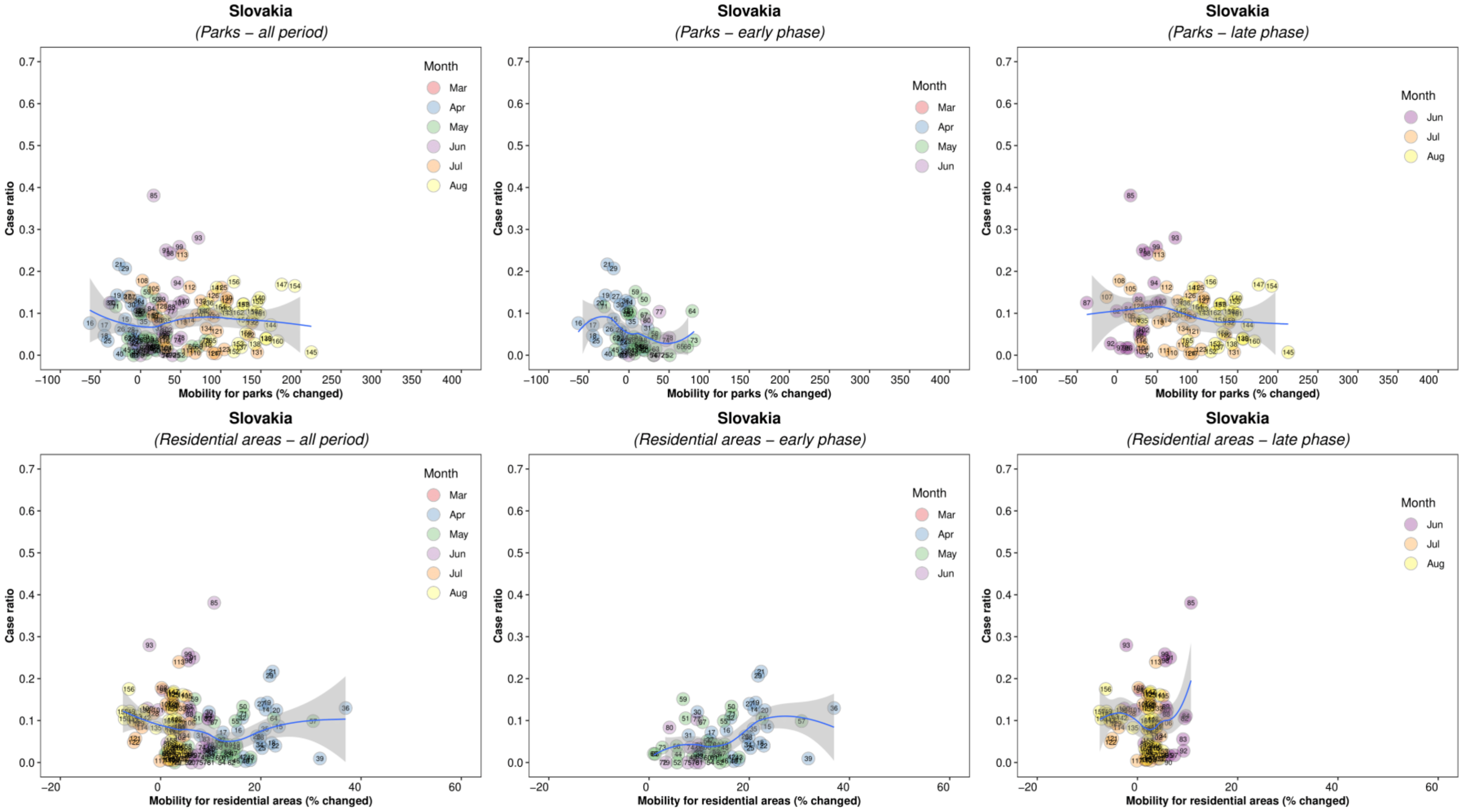
Slovakia
(Parks – all period)
Slovakia
(Parks – early phase)
Slovakia
(Parks – late phase)
Slovakia
(Residential areas – all period)
Slovakia
(Residential areas – early phase)
Slovakia
(Residential areas – late phase)
Case ratio
Mobility for parks (% changed)
Mobility for residential areas (% changed)
Month
Mar
Apr
May
Jun
Jul
Aug

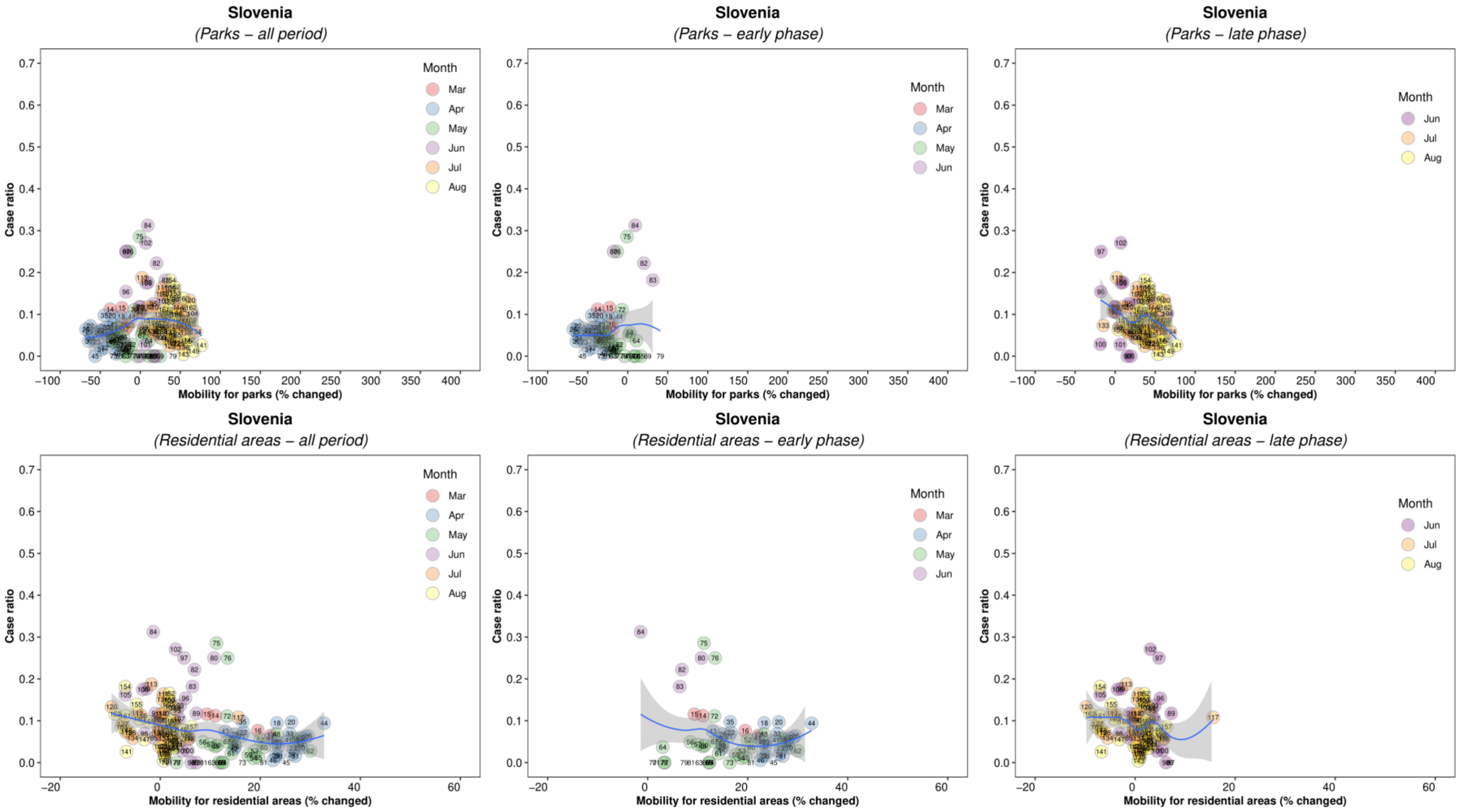
Slovenia
(Parks – all period)
Slovenia
(Parks – early phase)
Slovenia
(Parks – late phase)
Slovenia
(Residential areas – all period)
Slovenia
(Residential areas – early phase)
Slovenia
(Residential areas – late phase)
Case ratio
Mobility for parks (% changed)
Mobility for residential areas (% changed)
Month
Mar
Apr
May
Jun
Jul
Aug

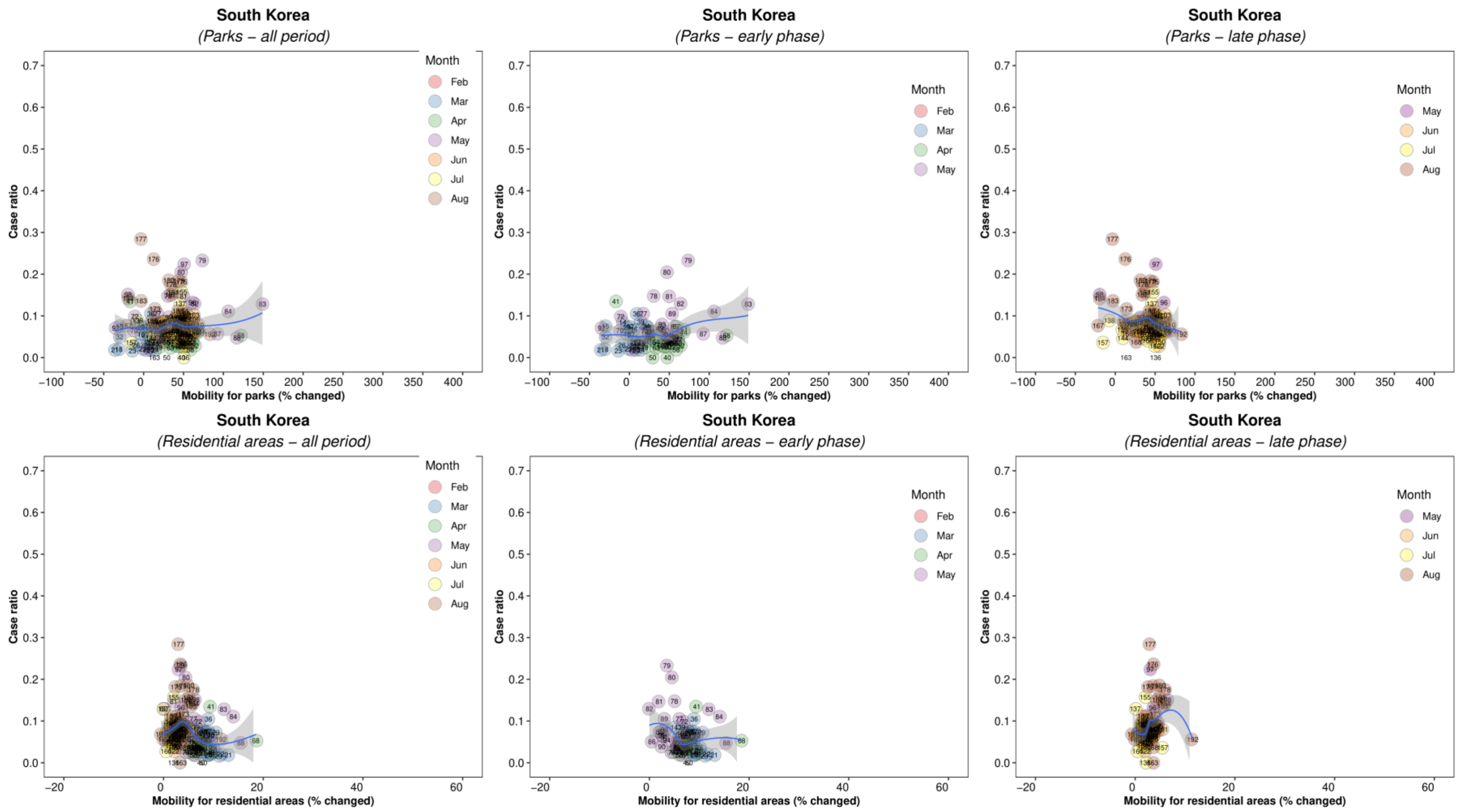
South Korea
(Parks – all period)
South Korea
(Parks – early phase)
South Korea
(Parks – late phase)
South Korea
(Residential areas – all period)
South Korea
(Residential areas – early phase)
South Korea
(Residential areas – late phase)
Case ratio
Mobility for parks (% changed)
Mobility for residential areas (% changed)
Month
Feb
Mar
Apr
May
Jun
Jul
Aug

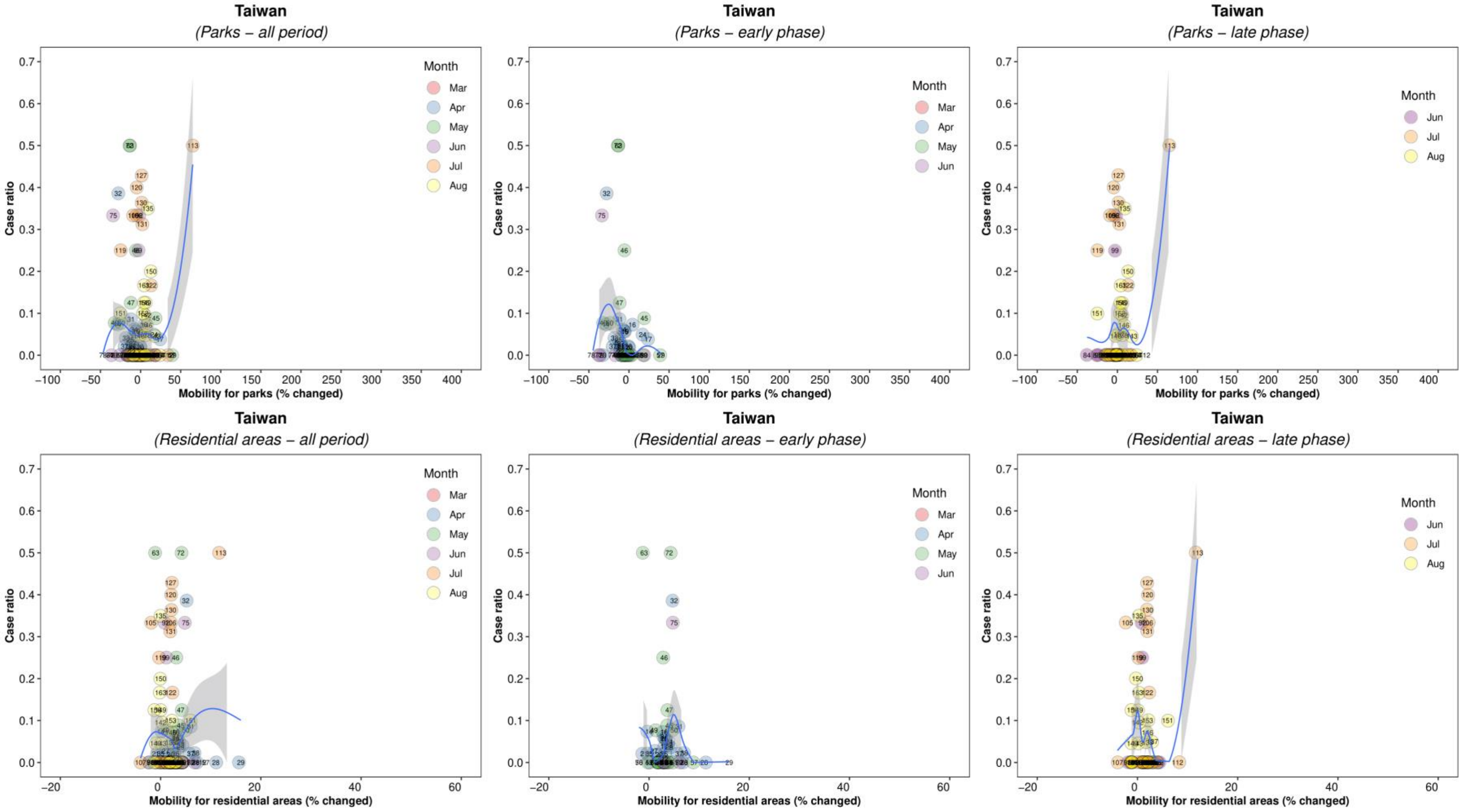
Taiwan
(Parks – all period)
Taiwan
(Parks – early phase)
Taiwan
(Parks – late phase)
Taiwan
(Residential areas – all period)
Taiwan
(Residential areas – early phase)
Taiwan
(Residential areas – late phase)
Case ratio
Mobility for parks (% changed)
Mobility for residential areas (% changed)
Month
Mar
Apr
May
Jun
Jul
Aug

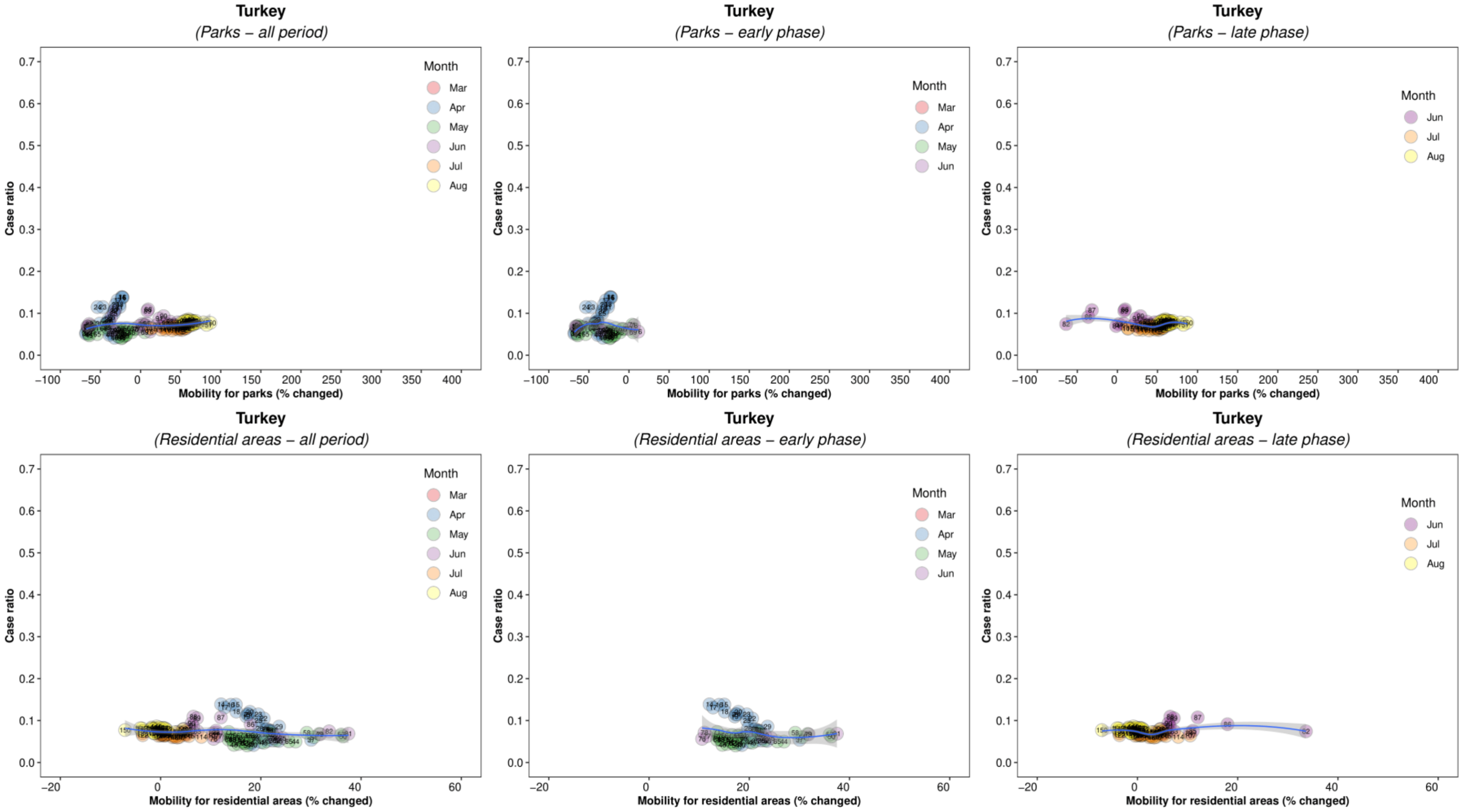
Turkey
(Parks – all period)
Turkey
(Parks – early phase)
Turkey
(Parks – late phase)
Turkey
(Residential areas – all period)
Turkey
(Residential areas – early phase)
Turkey
(Residential areas – late phase)
Case ratio
Mobility for parks (% changed)
Mobility for residential areas (% changed)
Month
Mar
Apr
May
Jun
Jul
Aug

Supplementary Figure 3. Association between new daily COVID-19 case ratio and mobility changes in 36 countries, for grocery and pharmacy visits

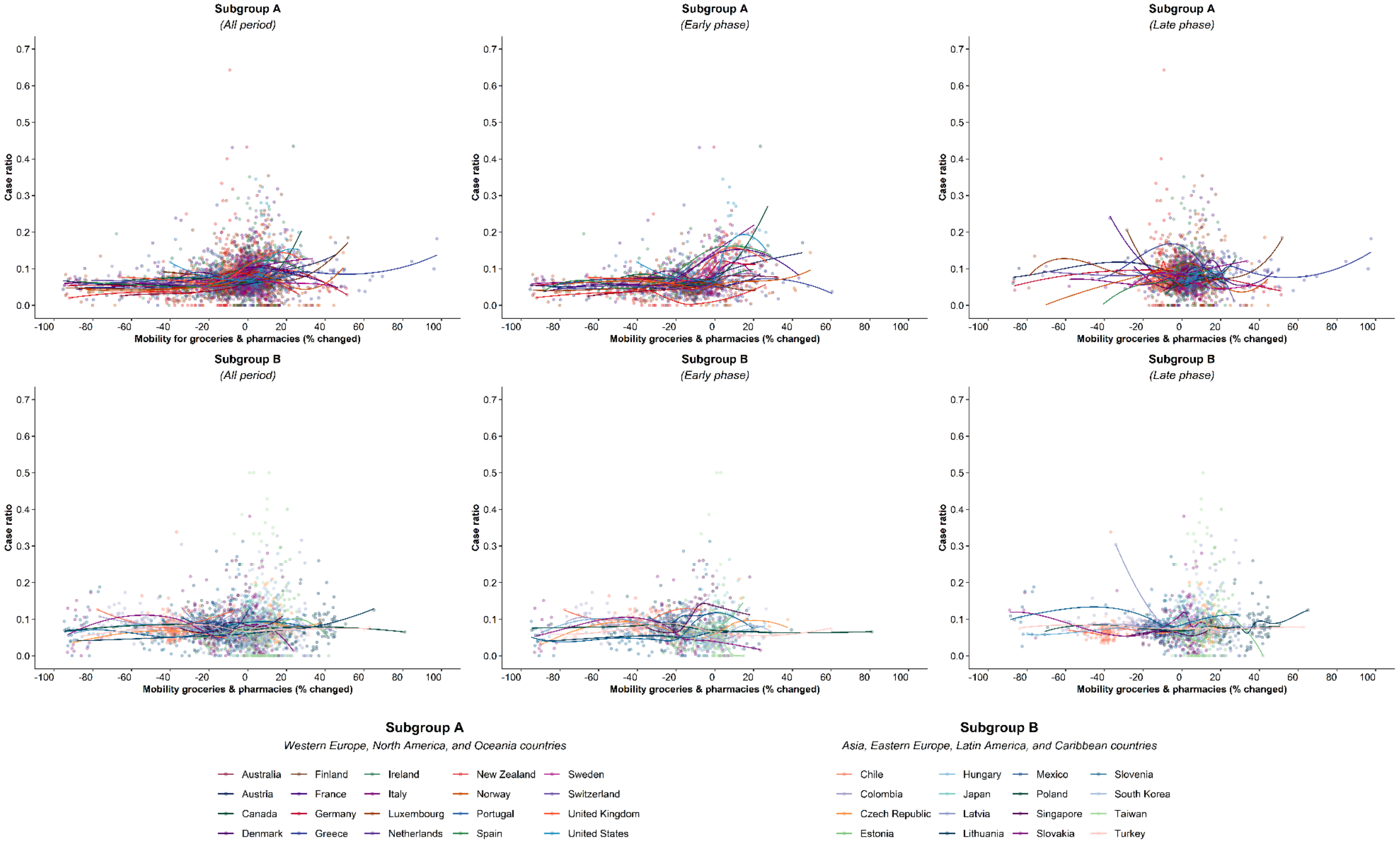

Footnote 1. The mobility change measurement period was from the day of the 100th case in each country through August 31, 2020.

Footnote 2. Pandemic phase was defined for each country by the median of the date when the 100th case was detected to the end of the study period: early phase for the period before the median date and late phase for the period after the median date

Supplementary Figure 4. Association between new daily COVID-19 case ratio and mobility changes for 36 countries based on alternative lag days

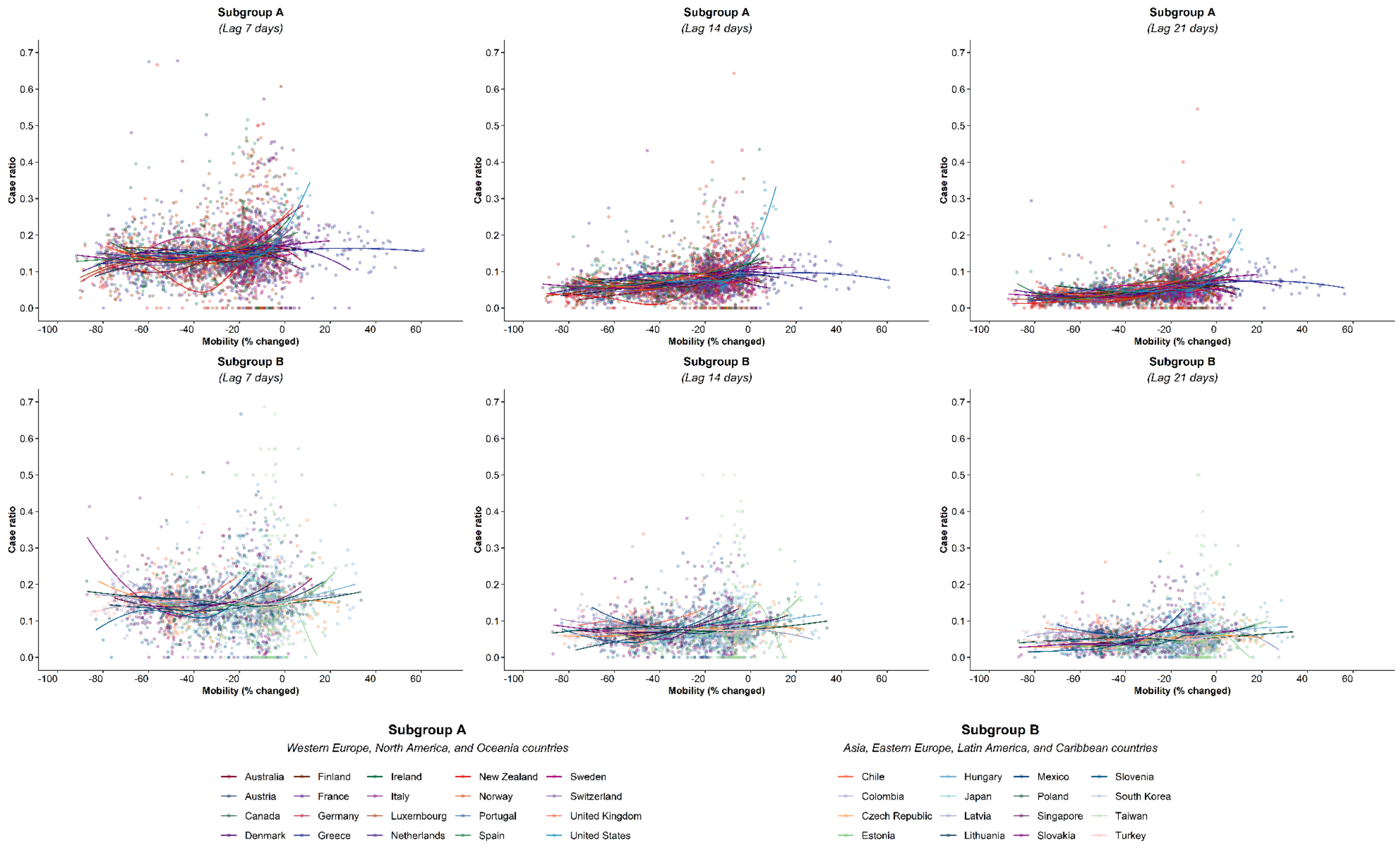

Footnote 1. The mobility change measurement period was from the day of the 100th case in each country through August 31, 2020.
